# Exploring the methodological quality and risk of bias in 200 systematic reviews: A comparative study of ROBIS and AMSTAR-2 tools

**DOI:** 10.1017/rsm.2025.10032

**Published:** 2025-10-27

**Authors:** Carole Lunny, Nityanand Jain, Tina Nazari, Melodi Kosaner-Kließ, Lucas Santos, Ian Goodman, Alaa A. M. Osman, Stefano Berrone, Mohammad Najm Dadam, Connor T. A. Brenna, Heba Hussein, Gioia Dahdal, Diana Cespedes A., Nicola Ferri, Salmaan Kanji, Yuan Chi, Dawid Pieper, Beverly Shea, Amanda Parker, Dipika Neupane, Paul A. Khan, Daniella Rangira, Kat Kolaski, Ben Ridley, Amina Berour, Kevin Sun, Radin Hamidi Rad, Zihui Ouyang, Emma K. Reid, Iván Pérez-Neri, Sanabel O. Barakat, Silvia Bargeri, Silvia Gianola, Greta Castellini, Sera Whitelaw, Adrienne Stevens, Shailesh B. Kolekar, Kristy Wong, Paityn Major, Ebrahim Bagheri, Andrea C. Tricco

**Affiliations:** 1 Knowledge Translation Program, St. Michael’s Hospital, Unity Health Toronto, Toronto, ON, Canada; 2 Anesthesiology, Pharmacology and Therapeutics, The University of British Columbia, Vancouver, BC, Canada; 3 Precision AQ, Vancouver, BC, Canada; 4 Independent Statistical Consultant, Chandigarh, India; 5 Department of Medical Geriatrics, School of Medicine, Tehran University of Medical Sciences, Tehran, Iran; 6 Independent Health Economics and Outcomes Research Specialist, Duisburg, Germany; 7 Masters and Doctoral Programs in Physical Therapy, Universidade Cidade de São Paulo, São Paulo, Brazil; 8 Deanery of Clinical Sciences, University of Edinburgh, Edinburgh, UK; 9 Department of Clinical Pharmacy and Pharmacy Practice, Faculty of Pharmacy, University of Gezira, Wad Madani, Sudan; 10 Department of Pharmacodynamics and Biopharmacy, Faculty of Pharmacy, University of Szeged, Szeged, Hungary; 11 Division of Dietetics, Nutrition and Biological Sciences, Physiotherapy, Podiatry and Radiography, School of Health Sciences, Queen Margaret University, Musselburgh, UK; 12 Department of Orthopedics and Trauma Surgery, Helios Klinikum Schwelm, Schwelm, Germany; 13 Department of Anesthesiology & Pain Medicine, University of Toronto, Toronto, ON, Canada; 14 Department of Physiology, University of Toronto, Toronto, ON, Canada; 15 Department of Oral Medicine and Periodontology, Faculty of Dentistry, Cairo University, Cairo, Egypt; 16 Department of Translational Medicine, University of Ferrara, Ferrara, Italy; 17 Department of Thoracic Surgery, The First Affiliated Hospital, Zhejiang University School of Medicine, Hangzhou, China; 18 Department of Biomedical and Neuromotor Sciences (DIBINEM), Alma Mater Studiorum, University of Bologna, Bologna, Italy; 19 The Ottawa Hospital and Ottawa Health Research Institute, Ottawa, ON, Canada; 20 Beijing Health Technology Co., Ltd, Beijing, China; 21 Department of Health Research Methods, Evidence, & Impact, McMaster University, Hamilton, ON, Canada; 22 Institute for Health Services and Health System Research, Faculty of Health Sciences Brandenburg, Brandenburg Medical School, Brandenburg, Germany; 23 Center for Health Services Research Brandenburg, Brandenburg Medical School, Brandenburg, Germany; 24 The Ottawa Health Research Institute, University of Ottawa, Ottawa, ON, Canada; 25 Knowledge Translation Program, Li Ka Shing Knowledge Institute, St. Michael’s Hospital, Toronto, ON, Canada; 26 Li Ka Shing Knowledge Institute, St. Michael’s Hospital, Unity Health Toronto, Toronto, ON, Canada; 27 Dalla Lana School of Public Health, University of Toronto, Toronto, ON, Canada; 28 Departments of Orthopaedic Surgery and Rehabilitation, Neurology, Pediatrics, and Epidemiology and Prevention, Wake Forest University School of Medicine, Winston Salem, NC, USA; 29 IRCCS Istituto delle Scienze Neurologiche di Bologna, Bologna, Italy; 30 University of Mouloud Maameri, Tizi Ouzou, Algeria.; 31 Department of Mechanical & Industrial Engineering, University of Toronto, Toronto, ON, Canada; 32 Faculty of Information, University of Toronto, Toronto, ON, Canada; 33 Department of Statistics, University of British Columbia, Vancouver, BC, Canada; 34 Department of Pharmacy, Nova Scotia Health Authority, Halifax, NS, Canada; 35 Evidence Synthesis Unit, National Institute of Rehabilitation Luis Guillermo Ibarra Ibarra, Mexico City, Mexico; 36 Faculty of Dentistry, Zarqa University, Zarqa, Jordan; 37 Unit of Clinical Epidemiology, IRCCS Istituto Ortopedico Galeazzi, Milan, Italy; 38 Faculty of Medicine and Health Sciences, McGill University, Montreal, QC, Canada; 39 Centre for Immunization Programs, Infectious Diseases and Vaccine Programs Branch, Public Health Agency of Canada, Ottawa, ON, Canada; 40 Consultant Adult Respiratory Medicine, Zealand University Roskilde Hospital, Roskilde, Denmark; 41 Department of Clinical Medicine, Clinical Care, Copenhagen university Hospital, Copenhagen, Denmark; 42 Clinical Care Group 1.01, European Respiratory Society, Lausanne, Switzerland; 43 School of Pharmacy, University of Waterloo, Waterloo, ON, Canada; 44 School of Nursing, McMaster University, Hamilton, ON, Canada; 45 Epidemiology Division and Institute of Health Policy, Management, and Evaluation, Dalla Lana School of Public Health, University of Toronto, Toronto, ON, Canada; 46 Queen’s Collaboration for Health Care Quality: A JBI Centre of Excellence, Queen’s University, Kingston, ON, Canada

**Keywords:** AMSTAR 2.0, bias, critical appraisal, quality, ROBIS, systematic reviews

## Abstract

AMSTAR-2 (A Measurement Tool to Assess Systematic Reviews, version 2) and ROBIS are tools used to assess the methodological quality and the risk of bias in a systematic review (SR). We applied AMSTAR-2 and ROBIS to a sample of 200 published SRs. We investigated the overlap in their methodological constructs, responses by item, and overall, percentage agreement, direction of effect, and timing of assessments. AMSTAR-2 contains 16 items and ROBIS 24 items. Three items in AMSTAR-2 and nine in ROBIS did not overlap in construct. Of the 200 SRs, 73% were low or critically low quality using AMSTAR-2, and 81% had a high risk of bias using ROBIS. The median time to complete AMSTAR-2 and ROBIS was 51 and 64 minutes, respectively. When assessment times were calibrated to the number of items in each tool, each item took an average of 3.2 minutes per item for AMSTAR-2 compared to 2.7 minutes for ROBIS. Nine percent of SRs had opposing ratings (i.e., AMSTAR-2 was high quality while ROBIS was high risk). In both tools, three-quarters of items showed more than 70% agreement between raters after extensive training and piloting. AMSTAR-2 and ROBIS provide complementary rather than interchangeable assessments of systematic reviews. AMSTAR-2 may be preferable when efficiency is prioritized and methodological rigour is the focus, whereas ROBIS offers a deeper examination of potential biases and external validity. Given the widespread reliance on systematic reviews for policy and practice, selecting the appropriate appraisal tool remains crucial. Future research should explore strategies to integrate the strengths of both instruments while minimizing the burden on assessors.

## Highlights

### What is already known?

- While systematic reviews (SRs) of intervention studies are used to support treatment recommendations, the methodological quality and risk of bias in reviews vary. AMSTAR-2 and ROBIS are tools designed to facilitate the critical appraisal of systematic reviews, with or without meta-analysis, terms of methodological quality and potential risks of meta-biases. Both Cochrane and JBI recommend that authors use ROBIS or AMSTAR-2 when comparing and critically appraising systematic reviews in the context of overviews of reviews or umbrella reviews.

### What is new?

- We found that 81% of SRs assessed had a high risk of bias, and 73% of SRs assessed with AMSTAR-2 were low or critically low methodological quality. The majority of items in the two tools overlapped fully or partially in content. Assessors reported faster assessment times with AMSTAR-2 compared to ROBIS. Three-quarters of items showed more than 70% agreement between senior and junior assessors in both tools after extensive training and piloting was conducted.
- A shorter median time was observed for AMSTAR-2 assessments than for ROBIS assessments (51 vs. 64 minutes). When the assessment times were calibrated to the number of items in each tool (16 items in AMSTAR vs. 24 items in ROBIS), the ROBIS timing was lower per minute than AMSTAR-2 (0.52 minutes faster).

### Potential impact for RSM readers

- The choice of instruments will depend on the user’s aim (i.e., methodological quality vs. a risk of bias assessment), the comprehensiveness of the assessment sought, whether external validity and bias in the conclusions are of interest, and other factors such as time constraints.

## 1 Background

The task of critically appraising research findings is crucial to informed decision-making in healthcare. Decision-makers such as policymakers, guideline developers, patients and their caregivers, and clinicians, rely on the highest quality studies to make decisions about which therapies, interventions, and policies should be implemented in real-world settings. The most reliable source of evidence to inform these decisions is systematic reviews (SRs), which summarise all relevant available evidence on a given topic. A 2022 survey by our team found that decision-makers frequently (98%) sought out SRs as a primary source of evidence.^1^ However, approximately 40% of decision-makers struggled to choose between the vast number of published SRs on a similar topic.^1^

Despite the importance of high-quality SRs, the concept of quality is not well defined in the literature. It can include constructs such as imprecision, reporting completeness, ethics, generalisability, and applicability.^2^ Importantly, a SR’s risk of bias is distinct from both its methodological quality (i.e., how well the review is conducted) and its reporting comprehensiveness or quality (i.e., how well the authors described their methodology and results). A risk of bias assessment focuses on the potential for study limitations to bias the review findings with respect to the topic of interest.

Bias in SRs occurs when factors systematically affect the results of a primary study or the review itself, producing a consistent deviation from the truth and potentially leading to inaccurate conclusions.^3^ Evaluation of SR-level biases (or meta-biases) relates to whether missing primary studies, analyses, or presented results can lead to over- or under-inflating the estimates of an intervention effect.^4^^–^ ^7^ These are often referred to as publication bias and/or other selective non-reporting biases. These concepts address situations in which a study, analyses, or results might not be reported for several reasons: (i) a study was performed but not published; (ii) the relevant result from an included study was not available to the SR authors; (iii) the SR authors had unintentionally failed to collect or process the data available; or (iv) the SR authors had intentionally excluded the result or an analysis from the SR. Missing or selectively omitting entire studies, specific findings such as outcome results, and unfavourable analyses within a SR can be influenced by factors such as the p-value of the result and the directionality or magnitude of the effect. Our definitions of methodological quality and risk of bias, along with other quality-related terms, are indexed in Box 1.

External assessors may be concerned with the potential risk of bias in the *results* of the SR, and/or the risk of bias in the *conclusions* drawn from the SR. The *results* refer to the set of quantitative estimates regarding the relative effects of interventions, while the *conclusions* pertain to the clinical and/or biological interpretations derived from the SR, which should account for all sources of uncertainty related to the results. SR authors may also ‘spin’ the interpretation of their results and mislead readers so that results are viewed in a more favourable light^8^^–^ ^12^ (Box 1).

Box 1. Definitions

*Systematic review*

A systematic review attempts to collate all study-specific evidence that fulfils pre-specified eligibility criteria to answer a specific research question. It uses explicit, systematic methods that are selected with a view to minimising bias, thus providing more reliable findings from which conclusions can be drawn and decisions made.^3^

*Pairwise meta-analysis*

Pairwise meta-analysis is a type of statistical synthesis, often used in systematic reviews, to combine effect estimates from primary studies (e.g., randomised controlled trials, cohort studies, case control studies) comparing one intervention with another.^3^

*Bias in results*

Bias occurs when factors systematically affect the results of a primary study or a systematic review and cause them to be different from the truth.^3^ The procedures that are required to conduct a meta-analysis (e.g., ensuring that studies are not selectively omitted) or the underlying systematic review (e.g., developing a comprehensive search strategy using multiple electronic databases and grey literature) help mitigate the risk of bias in the results.^13^ Studies affected by bias in the results can be inaccurate — particularly by over- or under-estimating the true effect in the target population.^13^

*Bias in conclusions*

A well-conducted systematic review draws conclusions that are appropriate to the evidence reviewed and can therefore be free of bias, even when the primary studies included in the review have a high risk of bias.^13^ However, bias can also be introduced when interpreting the review’s findings. For example, review conclusions may not be supported by the evidence presented, the relevance of the included studies may not have been considered by the review authors, and reviewers may inappropriately emphasise results based on their statistical significance alone.^13^

*Risk of bias*

Risk of bias is the likelihood that aspects of the design, conduct, analysis, interpretation, or reporting comprehensiveness of a study will lead to misleading results.^13^ A risk of bias assessment focuses on the potential for study limitations to skew the study findings with respect to the question of interest. It is distinguished from the methodological quality of studies (i.e., how well the study is conducted) and the reporting quality or comprehensiveness of a published evidence synthesis manuscript (i.e., how well authors report their methodology and results). ‘Risk of bias’ does not mean that the systematic review is decisively ‘biased’ or that the reviews themselves are not well-conducted.

*Types of instruments and assessments*

In systematic reviews, a domain-based *tool* refers to a structured instrument designed to assess specific aspects of bias and requires the reviewers to judge risk of bias or the methodological quality within specific domains, and to record the information on which each judgement was based (e.g., Cochrane RoB 2.0^14^).^15^ A *scale* is used to assess and numerically score studies based on various quality criteria (e.g., Jadad scale^16^). The score then allows for a composite score representing overall study quality.^15^^,^ ^17^ A *checklist* lists methodological criteria or questions that are used to assess studies without producing a score (e.g., Critical Appraisal Skills Programme checklists^18^).^17^

*Spin*

Spin is the use of misleading reporting strategies by authors to highlight a specific (e.g., positive) interpretation of the systematic review results if they were not in the intended direction or magnitude of effect, or if they were not statistically significant.^19^^,^ ^20^

*Applicability*

External validity consists of two unique underlying concepts—generalisability and applicability. Generalisability is about extending the results from a sample to the population from which the sample was drawn.^21^^,^ ^22^ Applicability is the extent to which the intervention effects observed are likely to reflect the expected results when a specific intervention is applied to the population of interest under ‘real-world’ conditions. A variety of terms have been used to describe applicability—directness, external validity, generalisability, and relevance.

GRADE approach to estimating the certainty in a body of evidence

In the Grading of Recommendations Assessment, Development and Evaluation (GRADE) framework, certainty of evidence reflects the degree of confidence that the estimated effect of an intervention or treatment used to support a decision or recommendation is close to the true effect. This assessment follows a structured process that takes into account factors such as risk of bias, inconsistency, indirectness, imprecision, and publication bias. According to the GRADE approach, four options can be chosen by the assessor to judge the certainty of evidence:

*(i) High certainty*

High-certainty evidence comes from well-conducted studies with consistent results and minimal risk of bias, and it is unlikely that further research will significantly change the confidence in the estimate.

*(ii) Moderate certainty*

Moderate certainty suggests that the available evidence is sufficient to support a conclusion, but further research may still impact the confidence in the estimate.

*(iii) Low certainty*

Low certainty implies that the available evidence is limited and the true effect may be substantially different from the estimate.

*(iv) Very low certainty*

Very low certainty indicates that the available evidence is insufficient to support any firm conclusions.

When external assessors want to determine if the conduct of an SR might bias its findings or conclusions, the ROBIS tool^23^ can be used (Box 2). A second tool called A Measurement Tool to Assess Systematic Reviews, version 2 (AMSTAR-2^24^) can be used to assess the methodological quality of the results of SRs (Box 2). Both tools are current and validated, with AMSTAR-2, in particular, seeing a wider adoption. This is reflected by the increased use of AMSTAR-2 over AMSTAR (version 1) in SRs and overviews of SRs,^25^ as well as the endorsement of both tools in the Cochrane Handbook of Systematic Reviews of Healthcare Interventions.^3^ The AMSTAR-2 checklist assesses whether the results of an SR of healthcare interventions have been well-conducted and consists of 16 items (seven critical and nine non-critical items). AMSTAR-2 lacks clear guidance on some items^26^^–^ ^28^ mandating the assessor to use their own judgement, which can lead to varying interpretations.

The ROBIS tool, on the other hand, assesses the risk of bias that may influence the SR findings and conclusions,^23^ and can be applied to intervention, diagnostic, aetiology, and prognostic SRs. ROBIS contains 21 items organised into four domains—domain 1: eligibility criteria (five items), domain 2: identification and selection of studies (five items), domain 3: data collection and study appraisal (five items), and domain 4: synthesis and findings (six items), in addition to domain-level (four items) and overall risk of bias judgments (three items). ROBIS has detailed instructions,^29^ which may require a substantially longer learning curve.^30^^–^ ^32^

Users of both the AMSTAR-2 and ROBIS tools require a high level of training,^3^^,^ ^26^ and the tools are often interpreted differently than their stated instructions.^31^ Therefore, the overall rating of both tools is likely vulnerable to an individual’s level of training, expertise in the SR topic being investigated, experience in applying each of the tools to SRs, and expertise in SR conduct, methods, and biases.^3^^,^ ^26^

Box 2. Comparison of AMSTAR-2 and ROBIS

**Table:** 

	AMSTAR-2	ROBIS
General characteristics		
Link	https://amstar.ca/Amstar-2.php	http://www.bristol.ac.uk/population-health-sciences/projects/robis/robis-tool/
Extent of user guidance	Extensive	Extensive
Clarity of user guidance	Allows for personal interpretation	Explicit with minimal room for personal interpretation
Review type	Intervention	Intervention, diagnostic, aetiology, prognostic^a^
Total number of items	16	21 plus 4 summarising items
Number of domains	None. 16 items categorised as critical (seven items) and non-critical (nine items)^b^	Four domains: 1. eligibility criteria (five items), 2. identification and selection of studies (five items), 3. data collection and study appraisal (five items), 4. synthesis and findings (six items)
Overall rating		
Construct	Level of methodological quality with seven items classified as ‘critical weaknesses’	Level of concern for risk of bias
Responses	High, moderate, low, and critically low	Low, high, unclear
Assessment focus	Results	Results and conclusions

Source: Adapted from Kolaski K, Logan LR, Ioannidis JPA. Guidance to best tools and practices for systematic reviews. JBI Evidence Synthesis 21(9):1699–1731, Sept. 2023. Notes: ^***a***^ROBIS includes an optional first phase to assess the applicability of the review to the research question of interest. The tool may be applicable to other review types in addition to the four specified, although modification of this initial phase will be needed (Personal Communication via email, Penny Whiting, University of Bristol, United Kingdom, dated 28 Jan. 2022). ^b^AMSTAR-2 item #9 and #11 requires separate responses for randomised controlled trials (RCTs) and non-randomised studies of interventions (NRSI).

Studies also demonstrate that the time spent using these tools ranged substantially (AMSTAR-2: 14–60 minutes and ROBIS: 16–60 minutes),^32^^–^ ^36^ not including time spent reading the SRs. The 14- to 60-minutes range is wide, and, in our experience, likely skewed towards the higher end of the time range. Furthermore, good practice requires two independent assessors to perform SR appraisal and then resolve any conflicts between their evaluations,^3^ which mandates additional time.

Of note, we use the term ‘assessment’ throughout this article, rather than ‘critical appraisal’. *Critical appraisal* is a broad term encompassing the evaluation of a study’s quality, including its risk of bias, relevance/applicability, the comprehensiveness of its reporting, as well as ethical considerations and issues such as imprecision. In contrast, *assessment* is a more focused term that distinguishes between evaluating methodological quality or risk of bias.

Herein, we applied both AMSTAR-2 and ROBIS to a cross-sectional sample of 200 SRs with and without meta-analysis. The objectives of this study were to: (a) map the items of both tools to compare their underlying constructs and identify item overlap; (b) determine the methodological quality and risk of bias in SRs using the AMSTAR-2 and ROBIS tools (i.e., overall and by item); (c) compare the time it takes to perform assessments with both tools; and (d) calculate the percentage agreement between assessors.

## 2 Methods

### 2.1 Study design

We followed SR guidance (i.e., Cochrane Handbook of Systematic Reviews of Healthcare Interventions^3^) for identification, study selection, and data extraction stages of our methodological study. We registered our protocol with the Open Science Framework (https://osf.io/nbcta/). To ensure comprehensive reporting, we adapted the Strengthening of Reporting of Observational Studies in Epidemiology for cross-sectional studies^37^ (Appendix A of the Supplementary Material).

### 2.2 Eligibility criteria

We included:

● SRs of observational epidemiological studies, such as those reporting prevalence and incidence information (henceforth called epidemiological SRs), or SRs investigating healthcare interventions.

● SRs with and without meta-analysis (e.g., pairwise meta-analysis, synthesis without meta-analysis);

● SRs defined as such by the authors (i.e., no definition was used for inclusion).

● SRs including any primary study design (e.g., randomised controlled trials (RCTs) and non-randomised studies of interventions [NRSI]); and

● SRs without restrictions on publication date or language.

We excluded overview of reviews (i.e., umbrella reviews or meta-reviews), SRs containing qualitative primary studies, methodological reviews, and scoping/evidence maps.

### 2.3 Dataset

To undertake this study, we leveraged two published methodological studies to collect their included SRs, in addition to conducting a search of the Cochrane Database of Systematic Reviews to identify SRs. These two methodological studies used systematic literature searches to locate SRs and contained full quality assessments of the SRs by the authors, using either the AMSTAR-2 or ROBIS tools. We then repurposed their quality assessments as the first independent assessment, akin to an independent external’s reviewer assessment. Our assessors then conducted a second, blinded, and independent assessment of these SRs using our decision rules (Section 2.6) and compared them to determine the percentage agreement. A total of 200 SRs and their assessments were retrieved from:

● 139 AMSTAR-2 assessed SRs from a methods study by Smires et al.^38^; and

● 34 ROBIS assessed SRs from a methods study by Banzi et al.^30^

In addition, a sample of 27 SRs was retrieved from the Cochrane Database of Systematic Reviews, from inception to September 14, 2023, using a licenced access to the database. Note that Smires et al.^38^ and Banzi et al.^30^ included Cochrane SRs, bringing the total number of Cochrane SRs in our sample to 68. The process we used to retrieve SRs from the Cochrane Database of Systematic Reviews is described in Appendix B of the Supplementary Material.

### 2.4 SR screening process

One reviewer, working independently, reviewed all titles/abstracts identified by the search, as well as the full texts of those citations deemed eligible for inclusion according to the Population, Interventions, Comparisons, Outcomes, and study design (PICOs) criteria. A second reviewer checked all citations, and any discrepancies were discussed until consensus was reached.

### 2.5 Crowdsourcing to recruit assessors

The Cochrane Engage website (https://engage.cochrane.org/tasks/3241) was used to crowdsource 27 volunteer assessors with experience in SR methods. The assessors were recruited from September 2023 to April 2024. To train the assessors, we sent them training materials via email as the first step in their remote self-training. We then either convened a virtual meeting or shared a recorded meeting, where we went over an example assessment and answered questions. The volunteers piloted a minimum of three SR assessments, which were checked by a senior reviewer. The work of the volunteer assessors was checked, and detailed feedback was given, until the full assessment complied with our decision rules (see Section 2.6), after which they were allowed to conduct assessments unchecked and provide guidance to other new assessors. After initial piloting, random checks were conducted to maintain 100% quality against our decision rules. WISEST assessors who had assessed 10 or more SRs and whose assessment were high quality could graduate to be quality checkers. Seven of the volunteer assessors graduated to quality checkers.

### 2.6 Decision rules

AMSTAR-2 and ROBIS require extensive training and are often interpreted differently than intended due to vague wording or broadly defined items with multiple components. This lack of clarity allows users significant leeway in their interpretation. For example, defining a ‘comprehensive search strategy’ (item 4 in AMSTAR-2) or an ‘appropriate range of databases’ (item 2.1 in ROBIS) is subjective and would likely be interpreted differently by different users. To address this, we developed decision rules to ensure consistent and standardised responses across the tools (Appendix C of the Supplementary Material). We also developed a clear set of instructions for how to extract data on the characteristics of the SRs.

To assess the synthesis sections of both tools, we needed to identify the primary intervention and outcome of interest. The primary outcome was extracted when it was explicitly defined in the title, abstract, objectives, introduction, or methods section. If the primary outcome was not found by this means, we extracted it as such from the reported power calculations or the first outcome mentioned in the manuscript. If multiple interventions were present, we chose the intervention/comparison describing the ‘experimental’ intervention versus placebo or standard of care for the primary outcome, or the first intervention highlighted in the title or abstract of the SR.

Assessors filled in the tool’s responses, along with a verbatim quote copied from the assessed SR to support the response judgement and a rationale for the quote and response entered. In addition, the assessor determined the clarity of the quote based on the following choices:

- Clear: Has a clear quote that makes the response judgement easy to make.
- Weak: (i) Has a quote, but it is vague or hard to understand; or (ii) has a quote, but the information is not complete. Missing components or more information needed; or (iii) has information in tables, supplements, or the protocol which does not include a quote from the SR manuscript or supplementary files.
- Difference: Has contradictory text—one quote says one thing, and another quote contradicts.
- No Information: No information in the full text, protocol, or supplements.

### 2.7 Data collection

We collected study characteristics and the PICOs eligibility criteria of each interventional SR, or Population, Exposure, Comparator, and Outcomes (PECO) eligibility criteria for non-intervention SRs. We determined whether SR authors assessed the certainty of the evidence using an appropriate tool (e.g., GRADE). We also considered whether authors gave a positive interpretation of the SR results even if they were not in the intended direction or magnitude of effect, or if they were not statistically significant (i.e., spin).^19^^,^ ^20^ Appendix D of the Supplementary Material contains a full list of data elements that were collected. Data were extracted from the SR manuscript, any web-based appendices or supplementary files available, and the publicly available protocol.

### 2.8 Quality check

AMSTAR-2 and ROBIS assessments were done by one assessor independently. A second senior assessor quality checked a proportion of the independently conducted assessments. Of the 200 assessed SRs, 84% (168/200) were checked by a senior assessor.

### 2.9 Data analysis

#### 2.9.1 Descriptive statistics

We summarised the characteristics of included SRs (e.g., number of authors, year) using descriptive statistics and presented the results in tables and figures. We reported central tendency and variability for continuous variables using the median and interquartile range (IQR) for skewed distributions, and the mean and standard deviation for symmetric distributions. We stratified SR characteristics by journal status (i.e., non-Cochrane vs. Cochrane) and provision of results from pairwise meta-analysis (i.e., Yes/No). Items from both tools reporting ‘Yes/Probably’, ‘Yes/Partial’, and ‘Yes’ responses were collapsed into Yes, while ‘No/Probably’ and ‘No’ responses were collapsed into No. Items with ‘No Information’ or that had a ‘Not applicable’ response (i.e., ROBIS item 4.5 when there was no meta-analysis conducted) were not counted towards the denominator but were noted in the Supplementary Material.

#### 2.9.2 Mapping AMSTAR-2 and ROBIS items for comparison

Each item in AMSTAR-2 and ROBIS was assessed based on its concept, approach, and definitions. This information was then used to match the items across the two instruments. A panel of five experts convened over several video meetings to map the items based on concept, approach, and description in each of the tools based on consensus methods. One researcher categorised the items based on whether they related to methodological quality, risk of bias, or reporting comprehensiveness (Box 1), which was checked by a second senior researcher. The same researchers determined whether the items in both tools related to relevance (applicability/external validity), that is, whether the PICOs/PECO of the included primary studies were similar to the PICOs/PECO of the SR authors’ targeted research question.

#### 2.9.3 Comparison of AMSTAR-2 and ROBIS ratings

For the assessment of methodological quality using AMSTAR-2, the 200 SRs were categorised as ‘High quality’, ‘Moderate quality’, ‘Low quality’, or ‘Critically low quality’. Notably, AMSTAR-2 wording states that the overall summary judgments range from ‘High confidence’ to ‘Critically low confidence’ in the results of the SR, as opposed to ‘High quality’.^24^ We have chosen to adopt the wording of ‘quality’ and not ‘confidence’, as AMSTAR-2 assesses methodological quality. This also avoids confusion with terminology used in the GRADE approach^39^ to rate the certainty of evidence across (vs. within) studies. In GRADE, there are four categories of certainty, which are based on the ‘level of confidence’ SR authors have that an effect estimate represents the true effect of an intervention (Box 1).

*Bias in results*. We rated ROBIS domains as ‘Low’, ‘High’, or ‘Unclear’ risk. If the answers to all signalling questions for a given domain were ‘Yes’ or ‘Probably Yes’, then the rating was Low risk of bias. The potential for bias was considered if any signalling question was answered ‘No’ or ‘Probably No’. For both tools, when an item was deemed ‘Not applicable’, it was not considered in the overall rating. For example, when a review did not conduct a quantitative analysis, all the items related to meta-analysis were rated as not applicable and were not counted towards the overall rating.

We compared the overall SR judgement ratings (i.e., High risk/Low quality vs. Low risk/High quality) of each of the tools and assessed when they disagreed in direction. We then examined qualitatively how the matched assessments diverged in direction of rating (i.e., Low vs. High) by item.

#### 2.9.4 Assessment time

For a balanced measurement of assessment time, we asked each assessor to alternate the order in which they applied the AMSTAR-2 and ROBIS tools for consecutive SRs. For example, the AMSTAR-2 was completed and timed for the first SR, then the ROBIS assessment was done and timed. For the second SR, the process was switched; ROBIS was completed first and timed, followed by an AMSTAR-2 assessment and its timing. This process ensured that the recorded times were accurate for each tool and that there was no memory effect on the assessment times.

We calculated the total time it took to assess each SR for AMSTAR-2 and ROBIS, and we standardised this estimate by dividing the number of questions on each tool. Recorded times account only for the time spent doing assessments (filling in the responses and adding quotes and rationale), and not for reading the SR or other ancillary tasks. We calculated the median time (in minutes) to complete one individual assessment and the IQR (due to the data being skewed) for each tool.

#### 2.9.5 Percentage agreement between assessors

When a pair of raters agreed or disagreed on a specific item, we recorded it as a raw percentage. Full agreement between the assessors was coded as 1, while no agreement was coded as 0, and a double hyphen was used to indicate missing assessment items. Percentage agreement was calculated using the proportion of times the raters agree without considering the possibility of chance agreement.

### 2.10 Deviations from protocol

We did not investigate the temporal changes in SR quality or risk of bias, as originally planned, using a stacked bar plot (stratified into before 31 December 2017 and after 1 January 2018). This was because a subset of 139 SRs (obtained from Smires et al.^38^) were published in 2017 (139/200; 69.5%), which would not have provided a representative sample for analysing prevalence over time. It would have also overstated the correlation between tool publication dates and quality improvement, without showing how quality fluctuated between other years.

## 3 Results

The study characteristics of the 200 SRs are presented in Table 1. Except for one SR in French, all other SRs were published in English. A total of 170 (85%) SRs were published prior to the broader adoption of the AMSTAR-2 tool, that is, prior to 31 December 2017, with 100 (50%) of these published in 2017. The remaining 30 (15%) SRs were published on or after 2018. The median number of authors was 5 (IQR 4, 6), and only one study, which was called an SR, had one author. The first authors were most frequently from Europe (66/200; 33%), North America (58/200; 29%), and Asia (50/200; 25%). Three topics—diseases of the skin and subcutaneous tissue, neoplasms, and diseases of the circulatory system—collectively accounted for 50% (100/200) of the SRs. Most were interventional SRs (146/200; 73%), with the remaining being epidemiological SRs (54/200; 27%). Two SRs were empty reviews, defined as SRs that found no studies eligible for inclusion.^40^ Pairwise meta-analysis of RCTs was the most common type of synthesis conducted (90/200; 45%), followed by narrative summary of RCTs/NRSI (68/200; 34%).

**Table 1:** Study characteristics of included systematic reviews (n = 200)

			Journal type	
Characteristic	Category	Total (n, %)	Cochrane (n = 68)	Non-Cochrane (n = 132)
ICD–10 medical classification	Diseases of the skin and subcutaneous tissue	45 (22.5%)	2 (2.9%)	43 (32.6%)
	Neoplasms	28 (14%)	8 (11.8%)	20 (15.2%)
	Diseases of the circulatory system	27 (13.5%)	5 (7.4%)	22 (16.7%)
	Diseases of the musculoskeletal system and connective tissue	27 (13.5%)	13 (19.1%)	14 (10.6%)
	Diseases of the respiratory system	15 (7.5%)	14 (20.6%)	1 (0.8%)
	Endocrine, nutritional, and metabolic diseases	12 (6.0%)	0 (0%)	12 (9.1%)
	External causes of morbidity and mortality	9 (4.5%)	7 (10.3%)	2 (1.5%)
	Symptoms, signs, and abnormal clinical and laboratory findings, not elsewhere classified	7 (3.5%)	4 (5.9%)	3 (2.3%)
	Injury, poisoning and certain other consequences of external causes	5 (2.5%)	5 (7.4%)	0 (0%)
	Factors influencing health status and contact with health services	6 (3.0%)	3 (4.4%)	3 (2.3%)
	Mental and behavioural disorders	6 (3.0%)	4 (5.9%)	2 (1.5%)
	Certain infectious and parasitic diseases	6 (3.0%)	1 (1.5%)	5 (3.8%)
	Diseases of the blood and blood-forming organs and certain disorders involving the immune mechanism	3 (1.5%)	0 (0%)	3 (2.3%)
	Diseases of the digestive system	1 (0.5%)	0 (0%)	1 (0.8%)
	Diseases of the ear and mastoid process	1 (0.5%)	1 (1.5%)	0 (0%)
	Diseases of the eye and adnexa	1 (0.5%)	1 (1.5%)	0 (0%)
	Diseases of the nervous system	1 (0.5%)	0 (0%)	1 (0.8%)
Number of authors per SR	1–3	47 (23.5%)	20 (29.4%)	27 (20.5%)
	4–6	107 (53.5%)	30 (44.1%)	77 (58.3%)
	7–9	33 (16.5%)	12 (17.6%)	21 (15.9%)
	≥10	13 (6.5%)	6 (8.8%)	7 (5.3%)
Number of authors per SR, median (IQR)	5 (4, 6)	5 (3, 7)	5 (4, 6)
Country of first authors	United States of America	43 (21.5%)	5 (7.4%)	38 (28.8%)
	China	28 (14.0%)	4 (5.9%)	24 (18.2%)
	United Kingdom	20 (10.0%)	15 (22.1%)	5 (3.8%)
	Australia	15 (7.5%)	10 (14.7%)	5 (3.8%)
	Canada	14 (7.0%)	7 (10.3%)	7 (5.3%)
	Denmark	12 (6.0%)	3 (4.4%)	9 (6.8%)
	Others (27 countries with seven or less SRs)	68 (34%)	24 (35.3%)	44 (33.3%)
Continent of first authors	Europe	66 (33.0%)	28 (41.2%)	38 (28.8%)
	North America	58 (29.0%)	13 (19.1%)	45 (34.1%
	Asia	50 (25.0%)	13 (19.1%)	37 (28%)
	Oceania	16 (8.0%)	11 (16.2%)	5 (3.8%)
	South America	8 (4.0%)	3 (4.4%)	5 (3.8%)
	Africa	2 (1.0%)	0 (0%)	2 (1.5%)
Year of publication	Prior to 31 December 2017	170 (85.0%)	49 (72.1%)	121 (91.7%)
	On or after 1 January 2018	30 (15.0%)	19 (27.9%)	11 (8.3%)
Type of SR synthesis	Pairwise meta-analysis of RCTs	90 (45.0%)	43 (61.8%)	47 (35.6%)
	Pairwise meta-analysis of NRSI	23 (11.5%)	0 (0%)	23 (17.4%)
	Pairwise meta-analysis of RCTs and non-RCTs	19 (9.5%)	4 (7.4%)	15 (11.4%)
	Narrative summary of RCTs or NRSI	68 (34.0%)	21 (30.9%)	47 (35.6%)
Type of SR	Intervention or treatment	146 (73.0%)	67 (98.5%)	79 (59.9%)
	Epidemiological (prevalence, incidence, aetiology)	54 (27.0%)	1 (1.5%)	53 (40.2%)
Number of included primary studies	0–4	29 (14.5%)	21 (30.9%)	8 (6.1%)
	5–9	52 (26.0%)	13 (19.1%)	39 (29.5%)
	10–14	33 (16.5%)	10 (14.7%)	23 (17.4%)
	15–19	22 (11.0%)	4 (5.9%)	18 (13.6%)
	20–29	25 (12.5%)	10 (14.7%)	15 (11.4%)
	30–39	15 (7.5%)	2 (2.9%)	13 (9.8%)
	≥ 40	24 (12.0%)	8 (11.8%)	16 (12.1%)
	Number of included primary studies, median (IQR)	12 (7, 25)	10 (4, 22)	14 (8, 26.5)
Certainty of evidence approach	Yes, GRADE used	66 (33%)	53 (77.9%)	13 (9.8%)
	Yes, non-GRADE approach used	5 (2.5%)	1 (1.5%)	4 (3.0%)
	No	129 (64.5%)	14 (20.6%)	115 (87.2%)
Certainty of evidence ratings (n = 71/200)	Very low certainty	23 (11.5%)	17 (25%)	6 (4.6%)
	Low certainty	22 (11.0%)	18 (26.5%)	4 (3.0%)
	Moderate certainty	17 (8.5%)	14 (20.6%)	3 (2.3%)
	High certainty	9 (4.5%)	5 (7.4%)	4 (3.0%)
	Not applicable (not evaluated)	129 (64.5%)	14 (20.6%)	115 (87.2%)
Funding	Not reported	32 (16.0%)	2 (2.9%)	30 (22.7%)
	Reported, no funding	60 (30.0%)	13 (19.1%)	47 (35.6%)
	Reported, institutional funding	83 (41.5%)	45 (66.2%)	38 (28.8%)
	Reported, private funding	15 (7.5%)	4 (5.9%)	11 (8.3%)
	Reported, combination of institutional and private funding	10 (5.0%)	4 (5.9%)	6 (4.6%)
Conflict of interests declared	Not reported	19 (9.5%)	3 (4.4%)	16 (12.1%)
	Reported, no conflict of interests	128 (64.0%)	43 (63.2%)	85 (64.4%)
	Reported, with conflict of interests	53 (26.5%)	22 (32.4%)	31 (23.5%)
Protocol	No protocol reported	116(58.0%)	7 (10.3%)	109 (82.6%)
	Yes, registered or published	57 (28.5%)	37 (54.4%)	20 (15.1%)
	Yes, mentioned in the manuscript but not registered, not published, or not retrievable	27 (13.5%)	24 (35.3%)	3 (2.3%)
Language	English	199 (99.5%)	68 (100%)	131 (99.2%)
	French (Translated to English)	1 (0.50%)	0 (0%)	1 (0.76%)
Equity	Yes	2 (1.00%)	2 (2.9%)	0 (0%)
	No	198 (99.0%)	66 (97.1%)	132 (100%)
Update	Yes	41 (20.5%)	37 (54.4%)	4 (3.0%)
	No	159 (79.5%)	31 (45.6%)	128 (97.0%)
Imprecision	No meta-analysis (i.e., not applicable)	60 (30.0%)	14 (20.6%)	46 (34.8%)
	Results are precise	50 (25.0%)	9 (13.2%)	41 (31.1%)
	Imprecision is likely	90 (45.0%)	45 (66.2%)	45 (34.1%)
Number of databases searched	1–2	36 (18.0%)	1 (1.5%)	35 (26.5%)
	3–4	86 (43.0%)	19 (27.9%)	67 (50.8%)
	5–6	47 (23.5%)	28 (41.2%)	19 (14.4%)
	≥7	31 (15.5%)	20 (29.4%)	11 (8.3%)
	Number of databases searched, median (IQR)	4 (3, 6)	5.5 (4, 7)	3 (2, 4)

Abbreviations: GRADE, Grades of Recommendation, Assessment, Development, and Evaluation; IQR, interquartile range; NSRI, non-randomised studies of interventions; RCTs, randomised controlled trials; SRs, systematic reviews.

### 3.1 Mapping AMSTAR-2 and ROBIS items for underlying constructs and overlap

ROBIS contains 24 items; five in domain 1, five in domain 2, five in domain 3, and six in domain 4, with an additional three items that aid in making an overall judgment of the risk of bias (i.e., items A, B, and C). AMSTSAR-2 contains 16 items. In addition, ROBIS contains a first phase where reviewers are invited to assess the external validity (generalisability/applicability of the findings), whereas AMSTAR-2 does not explicitly assess the generalisability or applicability of the findings. AMSTAR-2 primarily evaluates the methodological rigor and transparency of the SR process. While it includes items related to the framing and conduct of the SR (e.g., defining the research question, eligibility criteria, and reporting), it does not directly address whether the findings are generalisable or applicable to other settings or populations. The two tools had considerable overlap across their items after assessing the concept, approach, and definitions for each item (Table 2).

In some cases, one item from one instrument broadly encompassed two or more items from the other instrument (e.g., AMSTAR-2 item 4 encompassed ROBIS items 2.1, 2.2, 2.3, and 2.4). Table 2 shows how we mapped the ROBIS and AMSTAR-2 items, items that were not considered by the other instrument, and how we categorised the items based on bias, relevance, methodological quality, or reporting comprehensiveness. We judged items of both instruments as satisfactorily comparable with respect to concept, approach, and definitions, while in the case of one comparison (examination of publication bias/robustness of the results) we judged the items from the instruments as only partially overlapping (i.e., robustness of the SR/meta-analysis results includes an evaluation of publication bias as well as other considerations).

There were nine items in the ROBIS tool (items 1.2, 1.5, 2.4, 3.3, 3.5, 4.1, 4.2, B, and C) and three items in the AMSTAR-2 tool (items 7, 10, and 16) that did not sufficiently overlap in concept, approach, and description. Of the nine unique ROBIS items, three related to relevance or applicability of the included evidence to that of the SR question (items 1.2, 1.5, B), one item related to reporting comprehensiveness (item 1.5), three considered concepts related to bias in the selection of studies or publication bias (items 2.4, 4.1, 4.2), one item considered non-reporting bias (related to whether study data might be missed; item 3.3), one item related to the methodological quality (item 3.5), and a final item C considered bias in the conclusions of the SR. Item C considers bias in how the authors may have made a positive interpretation of the outcome’s effect estimates, even if not statistically significant, or may not have presented a balanced interpretation of all results (i.e., spin^19^^,^ ^20^). Among the three unique AMSTAR-2 items, three items related to reporting and methodological quality (items 7, 10, and 16), while item 7 additionally considered bias in the selection of studies.

**Table 2:** Mapping AMSTAR-2 and ROBIS items

		Item related to bias, quality,
AMSTAR-2 items	ROBIS items	reporting, or relevance
1. Did the research questions and inclusion criteria for the SR include the components of PICO?	1.3 Were eligibility criteria unambiguous?	Reporting and relevance
2. (i) Were methods established before the conduct of the SR and (ii) did the report justify any significant deviations from the protocol?	1.1 Did the SR adhere to predefined objectives and eligibility criteria?	Reporting and relevance
3. Did the SR authors explain their selection of the study designs for inclusion in the SR?	1.4 Were all restrictions in eligibility criteria based on date, sample size, study quality, outcomes appropriate?	Reporting and relevance
Not considered	1.2 Were the eligibility criteria appropriate for the SR question?	Relevance
Not considered	1.5 Were any restrictions in eligibility criteria based on sources of information appropriate (e.g., publication status or format, language, availability of data)?	Reporting and relevance
4. Did the SR authors use a comprehensive literature search strategy?	2.1 Did the search include an appropriate range of databases/ electronic sources for published and unpublished reports? 2.2 Were methods additional to database searching used to identify relevant reports? 2.3 Were the terms and structure of the search strategy likely to retrieve as many eligible studies as possible?	Reporting and bias in the selection of studies
Not considered	2.4 Were search strategy restrictions based on date, publication format, or language appropriate?	Bias in the selection of studies (related to whether studies might be missed)
5. Did the SR authors perform study selection in duplicate?	2.5 Were efforts made to minimise error in selection of studies?	Bias in the selection of studies (related to whether studies might be missed)
6. Did the SR authors perform data extraction in duplicate?	3.1 Were efforts made to minimise error in data collection?	Bias in the selection of studies (related to whether studies might be missed), and methodological quality (reliability)
7. Did the authors provide a list of excluded studies and justify the exclusions?	Not considered	Reporting and bias in the selection of studies
8. Did the authors describe the included studies in adequate detail?	3.2 Were sufficient study characteristics considered for both SR authors and readers to be able to interpret the results?	Reporting
Not considered	3.3 Were all relevant study results collected for use in the synthesis?	Non-reporting bias (related to whether data might be missing)
9. Did the authors use a satisfactory technique for assessing the risk of bias (RoB) in individual studies that were included in the SR?	3.4 Was risk of bias (or methodological quality) formally assessed using appropriate criteria?	Quality of conduct and primary study risk of bias
Not considered	3.5 Were efforts made to minimise error in risk of bias assessment?	Methodological quality (reliability)
Not considered	4.1 Did the synthesis include all studies that it should?	Bias in the selective reporting of studies (related to whether study data might be missing)
Not considered	4.2 Were all pre-defined analyses reported or departures explained?	Bias in the selective reporting of analyses
11. If meta-analysis was performed did the SR authors use appropriate methods for statistical combination of results?	4.3 Was the synthesis appropriate given the nature and similarity in the research questions, study designs and outcomes across included studies?	Quality of conduct
14. Did the SR authors provide a satisfactory explanation for, and discussion of, any heterogeneity observed in the results of the SR?	4.4 Was between-study variation (heterogeneity) minimal or addressed in the synthesis?	Quality of conduct
15. If they performed quantitative synthesis did the SR authors carry out an adequate investigation of publication bias (small study bias) and discuss its likely impact on the results of the SR?	4.5 Were the findings robust, e.g., as demonstrated through funnel plot or sensitivity analyses?	Publication bias and/or other selective non-reporting biases
12. If meta-analysis was performed, did the SR authors assess the potential impact of RoB in individual studies on the results of the meta-analysis?	4.6 Were biases in primary studies minimal or addressed in the synthesis?	Primary study risk of bias
10. Did the SR authors report on the sources of funding for the studies included in the SR?	Not considered	Reporting and methodological quality
16. Did the SR authors report any potential sources of conflict of interest, including any funding they received for conducting the SR?	Not considered	Reporting and methodological quality
(Partial: 13. Did the SR authors account for RoB in individual studies when interpreting/discussing the results of the SR?)	A. Did the interpretation of findings address all of the concerns identified in Domains 1–4?	Acknowledgement by the SR authors of any meta-biases introduced during its conduct
Not considered	B. Was the relevance of identified studies to the SR’s research question appropriately considered?	Relevance
Not considered	C. Did the authors of the SR avoid emphasising results on the basis of their statistical significance?	Spin/Selective reporting

*Abbreviations**:*** RoB, risk of bias; PICO, population, intervention, comparator and outcome; SR, systematic review.

### 3.2 Comparison of overall judgments and direction of ratings

A comparison of the overall judgement of the 200 SRs for both tools is found in Appendix E of the Supplementary Material. Our 200 ROBIS assessments indicated that 162 (81%) SRs had a high risk of bias, with the remaining 38 (19%) considered to be low risk. Similarly, our 200 AMSTAR-2 assessments indicated that the majority of SRs (146/200; 74%) were considered either low (21/200 [10.5%]) or critically low quality (125/200 [62.5%] critically low). Only 39 (19.5%) of those SRs were considered high quality, and 15 (7.5%) were moderate quality.

Using ROBIS, we found that 37 out of 68 (54.4%) Cochrane SRs were deemed to be at low risk of bias. Comparatively, using AMSTAR-2, we found that most Cochrane SRs (38/68 [55.9%]) were high quality, and 14/68 (20.6%) were moderate quality. Among the 132 non-Cochrane SRs, we found that only one (0.8%) was deemed to be at low risk of bias using ROBIS. Using the AMSTAR-2 checklist, only one (0.8%) SR was high quality, and one (0.8%) was moderate quality. Of note, the three low risk/higher quality non-Cochrane SRs assessed using the two tools were different studies (Appendix E of the Supplementary Material).

When we consider the 132 SRs that conducted meta-analysis, ROBIS assessments indicated that 25/132 (18.9%) were low risk, and a similar number were high quality (26/132; 19.1%), and moderate quality (13/132; 9.8%) using AMSTAR-2. Of the 68 SRs without meta-analysis, 13/68 (17.6%) were low risk using ROBIS, and a similar number (13/68; 17.6%) were high quality, and two (2.9%) were classified as moderate quality using AMSTAR-2.

### 3.3 Comparison of ROBIS and AMSTAR-2 item ratings

**Figure 1:**
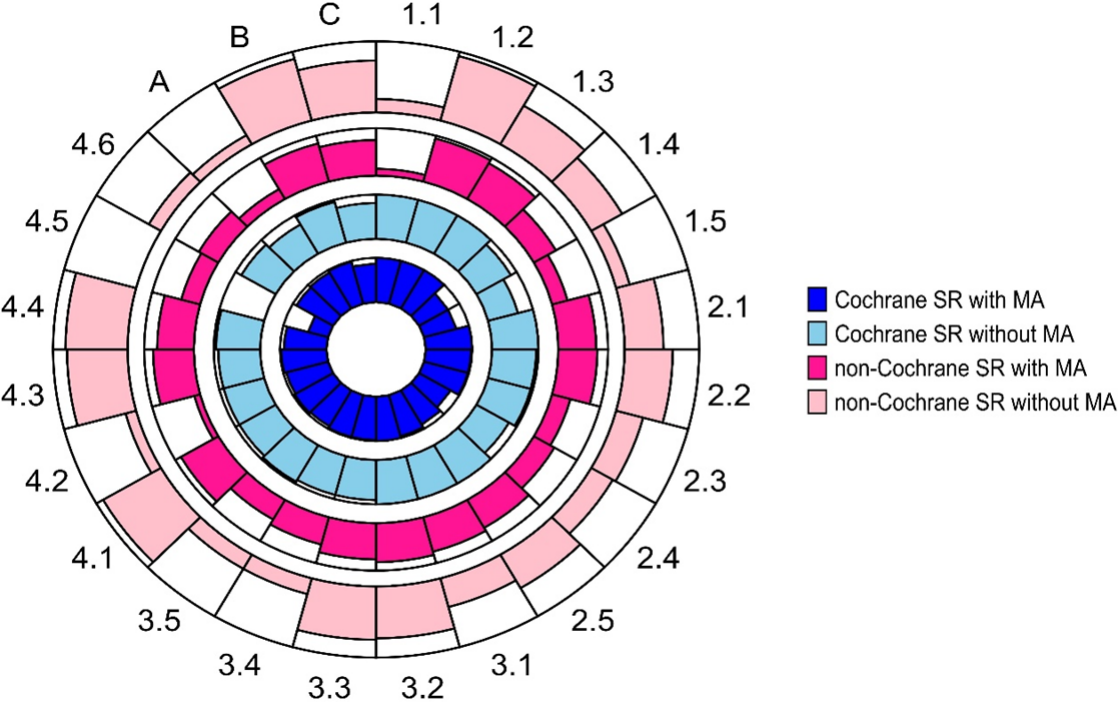
Circular bar plot showing the proportion of ROBIS items assessed positively (‘Yes’ or ‘Probably Yes’) across Cochrane and non-Cochrane systematic reviews (SRs), stratified by with or without meta-analysis (n = 200). Navy blue colour represents Cochrane SRs with MA; Teal colour represents Cochrane SRs without MA; Fusia colour represents non-Cochrane SRs with MA; and Pink respresents non-Cochrane SRs without MA. Bar height reflects the percentage of ROBIS items assessed positively (0–100% scale). ROBIS item description is provided in Table 2. Abbreviations: MA, meta-analysis; SR, systematic review.

**Figure 2:**
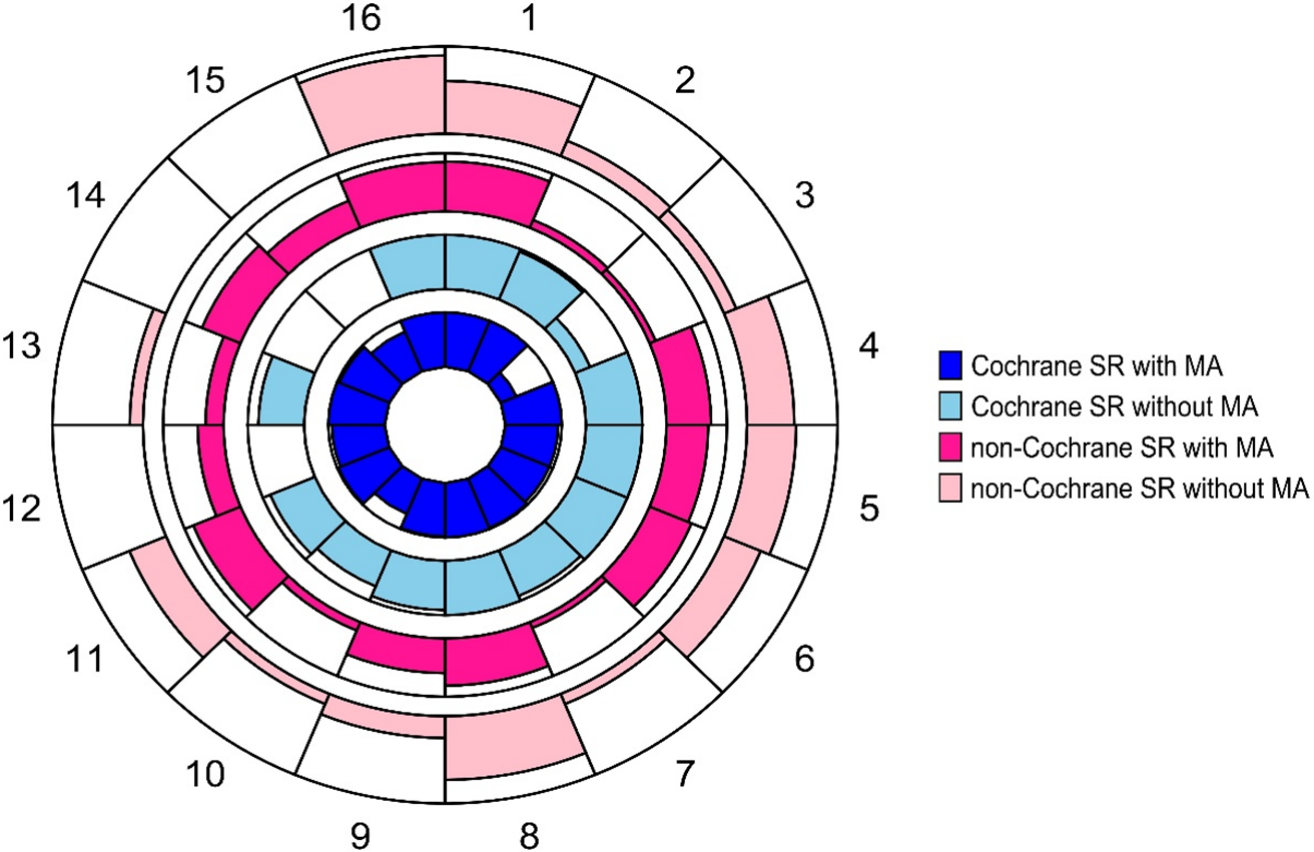
Circular bar plot showing the proportion of AMSTAR-2 items assessed positively (‘Yes’ or ‘Probably Yes’) across Cochrane and non-Cochrane systematic reviews (SRs), stratified by with or without meta-analysis (n = 200). Navy blue colour represents Cochrane SRs with MA; Teal colour represents Cochrane SRs without MA; Fusia colour represents non-Cochrane SRs with MA; and Pink respresents non-Cochrane SRs without MA. Bar height reflects the percentage of AMSTAR-2 items assessed positively (0–100% scale). AMSTAR-2 item description is provided in Table 2. Abbreviations: MA, meta-analysis; SR, systematic review.

Figures 1 and 2 show items reported positively (Yes/Probably Yes/Partial Yes) for each of the tools, stratified by Cochrane and non-Cochrane SRs, and SRs with and without meta-analysis. Tabular representation of the assessment ratings supporting the figures is presented in Tables F.1 and F.2 in the Supplementary Material. The majority of matched ROBIS and AMSTAR-2 items achieved (near) similar responses. For example, when considering all 200 SRs, ROBIS item 1.1 and AMSTAR-2 item 2, both dealing with the existence of a pre-defined protocol, were assessed positively in 44.5% (89/200) and 44% (88/200) SRs, respectively.

The majority of Cochrane SRs *with* meta-analysis had high quality/low risk of bias responses ranging from 91.5% to 100.0% for ROBIS items, and from 93.6% to 100% for AMSTAR-2 items. A relatively lower positive response rate in Cochrane SRs *with* meta-analysis (~75%) was observed for ROBIS items 1.4, 1.5, 2.4, 4.5, and AMSTAR-2 items 10 and 15. Of concern, a very low positive response rate was found for AMSTAR-2 item 3 at 29.8% (i.e., ‘Did the SR authors explain their selection of the study designs for inclusion in the SR?’). Similarly, the majority of Cochrane SRs *without* meta-analysis had high positive responses from 90.5% to 100% for most of the ROBIS and AMSTAR-2 items, with relatively lower positive responses (<75%) found for ROBIS items 1.5 and A, and AMSTAR-2 items 10 (67%) and 3 (29%).

Opposite trends were observed in non-Cochrane SRs as compared to Cochrane SRs. Only ROBIS item 1.2 achieved similarly high positive responses compared to Cochrane SRs (range 93.6–95.3%). Many non-Cochrane SRs *with* meta-analysis and those *without* meta-analysis achieved positive responses of less than 90%, ranging from 10.6% to 89.4% for most ROBIS and AMSTAR-2 items, which were also lower than the positive Cochrane SRs responses. Additionally, lower positive responses were observed for non-Cochrane SRs *with* meta-analysis compared to Cochrane SRs for eight ROBIS items (items 1.1, 1.5, 2.3, 2.4, 3.5, 4.2, 4.6, A) and seven AMSTAR-2 items (items 2, 3, 7, 10, 12, 13, and 15). For example, ROBIS item 1.1 about the existence of a protocol was only rated positive for 14.3% non-Cochrane SRs *with* meta-analysis, compared to their Cochrane counterparts at 100%, and similarly for AMSTAR-2 item 2 (i.e., 14.1% vs. 100%, respectively).

### 3.4 Direction of ratings

In total, 18 SRs (9%) had a ROBIS and AMSTAR-2 overall judgement that were discordant in the direction of ratings (i.e., high risk/high or moderate quality, low risk/low or critically low quality). From the six AMSTAR-2 critical items (i.e., items 2, 9, 11, 13, and 15) which matched to a ROBIS item (i.e., items 1.1, 4.3, A, 4.5 respectively), 15/18 (83.3%) SRs were judged as low risk/high quality (i.e., they were judged as ‘Yes’ for these 18 SRs). Therefore, in the majority of cases, these six items did not lead to a discordant direction of rating between the two instruments. The discrepancy in direction of rating occurred due to unique items in the ROBIS and AMSTAR-2 tools (i.e., non-matched items showed discrepancies). Notably of the 18 SRs, AMSTAR-2 item 3 was not reported by 12/18 (66.7%) SR authors (‘Did the SR authors explain their selection of the study designs for inclusion in the SR’), and 10/18 (55.6%) authors did not report on the restrictions in eligibility criteria (ROBIS items 1.4 and 1.5). Then, seven of the 18 (38.9%) SR authors did not report on the sources of funding for the studies included in the SR (AMSTAR-2 item 10).

### 3.5 Percentage agreement between assessors

The percentage agreement between any two assessors is found in Table 3 for the 166 SRs (83%) that were checked. Of the 28 items in total across the two tools, only seven (25%) were below 70% agreement. All items rated as below 70% agreement were matched during our ROBIS/AMSTAR-2 item mapping exercise (Section 3.1). Then, 7 of the 28 items (25%) had over 90% agreement between assessors. No item fell below 59.4% agreement.

**Table 3:** Percentage agreement of ROBIS assessments

ROBIS	AMSTAR-2	Percentage agreement (%)
1.1 Did the SR adhere to pre-defined objectives and eligibility criteria?	2. Were SR methods established prior to the conduct of the SR and did the report justify any significant deviations from the protocol?	128/166 (77.1)
1.2 Were the eligibility criteria appropriate for the SR question?	Not considered	120/130 (92.3)
1.3 Were eligibility criteria unambiguous?	1. Did the research questions and inclusion criteria for the SR include the components of PICO?	101/164 (61.6)
Not considered	3. Did the SR authors explain their selection of the study designs for inclusion in the SR?	119/133 (88.1)
1.4 Were all restrictions in eligibility criteria based on study characteristics appropriate (e.g., date, sample size, study quality, outcomes measured)?	Not considered	103/130(79.2)
1.5 Were any restrictions in eligibility criteria based on publication status or format, language, availability of data appropriate?	Not considered	114/129 (88.4)
2.1 Did the search include an appropriate range of databases/ electronic sources for published and unpublished reports?	4. Did the SR authors use a comprehensive literature search strategy?	98/165 (59.4)
2.2 Were methods additional to database searching used to identify relevant reports?	Not considered	119/130 (91.5)
2.3 Were the terms and structure of the search strategy likely to retrieve as many eligible studies as possible?	Not considered	112/129 (86.8)
2.4 Were search strategy restrictions based on date, publication format, or language appropriate?	Not considered	105/131 (80.2)
2.5 Were efforts made to minimise error in selection of studies?	5. Did the SR authors perform study selection in duplicate?	118/125 (71.5)
3.1 Were efforts made to minimise error in data collection?	6. Did the SR authors perform data extraction in duplicate?	107/165 (64.8)
Not considered	7. Did the SR authors provide a list of excluded studies and justify the exclusions?	112/132 (84.8)
3.2 Were sufficient study characteristics considered for both SR authors and readers to be able to interpret the results?	8. Did the SR authors describe the included studies in adequate detail?	118/165 (71.5)
3.3 Were all relevant study results collected for use in the synthesis?	Not considered	120/129 (93.0)
3.4 Was risk of bias (or methodological quality) formally assessed using appropriate criteria?	9. Did the SR authors use a satisfactory technique for assessing the risk of bias (RoB) in individual studies that were included in the SR?	113/165 (68.5)
3.5 Were efforts made to minimise error in risk of bias assessment?	Not considered	131/144 (91.0)
4.1 Did the synthesis include all studies that it should?	Not considered	121/130 (93.1)
4.2 Were all pre-defined analyses reported or departures explained?	Not considered	114/129 (88.4)
4.3 Was the synthesis appropriate given the nature and similarity in the research questions, study designs and outcomes across included studies?	Partial: 11. If meta-analysis was performed did the SR authors use appropriate methods for statistical combination of results?	114/163 (69.9)
4.4 Was between-study variation (heterogeneity) minimal or addressed in the synthesis?	14… ., any heterogeneity observed in the results of the SR?	110/164 (67.1)
Partial: 4.5 Were the findings robust, e.g., as demonstrated through funnel plot or sensitivity analyses?	15. If they performed quantitative synthesis did the SR authors carry out an adequate investigation of publication bias (small study bias) and discuss its likely impact on the results of the SR?	122/163 (74.8)
4.6 Were biases in primary studies minimal or addressed in the synthesis?	12. If meta-analysis was performed, did the SR authors assess the potential impact of RoB in individual studies on the results of the meta-analysis?	120/164 (73.2)
Not considered	10. Did the SR authors report on the sources of funding for the studies included in the SR?	112/130 (86.2)
Not considered	16. Did the SR authors report any potential sources of conflict of interest, including any funding they received for conducting the SR?	122/134 (91.0)
A. Did the interpretation of findings address all of the concerns identified in Domains 1–4?	13 … account for RoB when interpreting/ discussing the results of the SR?	106/164 (64.6)
B. Was the relevance of identified studies to the SR’s research question appropriately considered?	Not considered	119/128 (93.0)
C. Did the reviewers avoid emphasising results on the basis of their statistical significance?	Not considered	110/127 (86.6)

*Abbreviation**:*** RoB, risk of bias.

### 3.6 Assessment time

Assessment time for two consecutive SRs was reported by 14 out of 27 (52%) reviewers. A total of 53/61 (87%) assessments were timed using both AMSTAR-2 and ROBIS, while eight (13%) were timed using only one of these tools. Many assessors provided assessment time from multiple SRs, with one assessor providing data for 22 SRs. The median time to complete AMSTAR-2 assessments was 51 minutes (IQR 26–67 minutes) when applied first and decreased to 15 minutes (IQR 10–40 minutes) when applied after the ROBIS tool. Median time to complete ROBIS assessments was 64 minutes (IQR 55–77 minutes) when applied first and decreased to 53 minutes (IQR 41–64) when applied after the AMSTAR-2 tool.

When the assessment times were calibrated to the number of items in each tool (16 items in AMSTAR vs. 24 items in ROBIS), the ROBIS timing was lower per minute than AMSTAR-2 (0.52 minutes faster). Specifically, an average of 3.19 minutes per item was recorded for AMSTAR-2 when it was applied first, compared to 2.67 minutes per item for ROBIS when it was applied first. The longest assessment times were reported by two assessors who had completed no more than three prior assessments and had limited to no familiarity with the tools.

**Figure 3:**
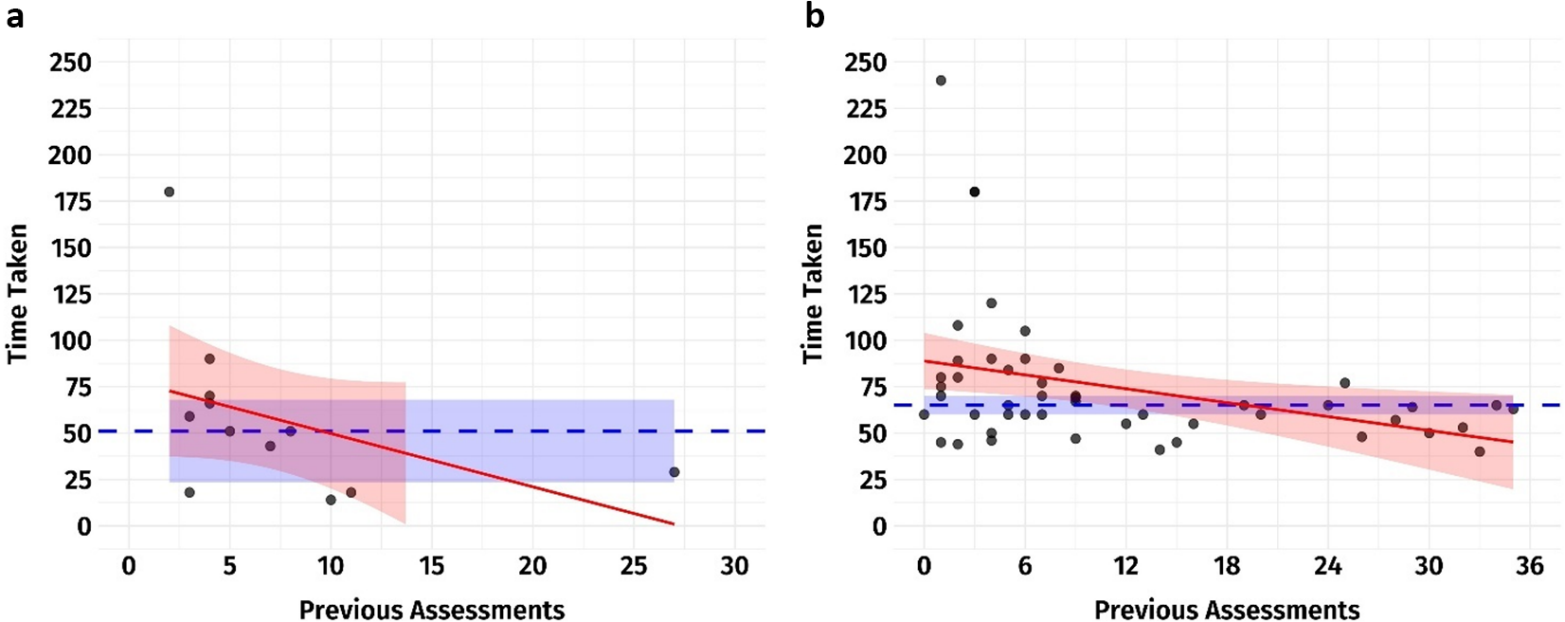
Total assessment time, in minutes, for (a) AMSTAR-2 when AMSTAR-2 is applied first; and (b) for ROBIS when ROBIS is applied first, depending on the number of previous assessments performed. Each dot represents an individual’s timing, with the median (dashed blue line) and its 95% confidence interval (blue shaded area), as well as the overall trendline (red line), and its standard error (red shaded area). The confidence interval for the median was calculated by bootstrapping for n = 1,000 samples.

When AMSTAR-2 was applied for the first time, the time required for each individual assessment reached a plateau after the assessor had completed five previous assessments, falling below the median of 51 minutes (95% CI bootstrap: 24–68 minutes). When ROBIS was applied first, the time required per individual assessment reached a plateau after 12 previous assessments were completed, stabilising at a value that was, in most cases, closer to or below the median of 65 minutes (95% CI bootstrap: 60–70 minutes). This suggests that user familiarity was achieved considerably more rapidly with AMSTAR-2 than with ROBIS tools (Figure 3).

## 4 Discussion

Our methodological study provides a comprehensive assessment of the quality and risk of bias assessment of 200 SRs across a range of biomedical fields using the AMSTAR-2 and ROBIS tools. These two tools are used to assess SR-level methodological quality and biases (i.e., meta-biases) that may occur when reviews synthesise evidence from primary studies. Guidance documents (e.g., Cochrane^41^ and JBI^42^) recommend that authors use ROBIS or AMSTAR-2 when comparing and critically appraising SRs in the context of conducting overviews of reviews. In terms of efficiency, our assessors took a median of 51 minutes to assess each SR using AMSTAR-2 and 64 minutes when using ROBIS, when these tools were applied first in order.

### 4.1 Mapping items for potential overlap in quality and meta-biases

We found that both tools had significant overlap in the content of the items. However, the nine unique items in ROBIS, and the three unique items in AMSTAR-2, means that results from the two tools cannot be directly compared. Indeed, we found that these unique items meant that assessment ratings were found in opposite directions in 9% of cases for the same SRs (i.e., ROBIS was high risk while AMSTAR-2 was high quality). When reviewers need to choose one of the tools based on construct, ROBIS may be better when a SR’s conclusion needs to be assessed for bias and spin, and when the generalisability of the findings needs to be considered. Additionally, ROBIS is specifically designed to detect bias in the results and conclusions, whereas AMSTAR-2 focuses on the SR’s methodological quality (providing details about reporting comprehensiveness, methodological quality, and bias).

### 4.2 Overall ratings of the methodological quality and risk of bias in SRs

Most SRs from the analysed dataset were at high risk of bias and of low methodological quality, as measured by the ROBIS and AMSTAR-2 tools, respectively. A higher risk of bias and lower quality was identified in SRs without meta-analysis, and in non-Cochrane SRs. Our findings are consistent with other studies, which generally show that AMSTAR-2 ratings are consistent with the overall risk of bias ratings in ROBIS.^32^^,^ ^34^ However, our study reported lower rates for critically low or low quality for SRs using AMSTAR-2 (73%) and a high risk of bias using ROBIS (81%) compared to other studies with similar characteristics^43^^–^ ^47^ (Box 3). These empirical studies assessing SRs on a variety of healthcare topics reveal that problems with SR quality/bias are not limited to medical field specialties (e.g., chemotherapy, nutrition), certain review types (e.g., intervention vs. epidemiological), and type of included primary study designs (RCT vs. NRSI), as can be seen in Box 3.^48^

Box 3. Previous studies assessing systematic reviews using both AMSTAR-2 and ROBIS

**Table:** 

Study	Topic	Number of SRs assessed	Dates of SRs	AMSTAR-2 low/critically low-quality rating	ROBIS high risk of bias rating
Zajac 2022^47^	Nutrition	101	2010–2018	97%	97%
Storman 2020^46^	Bariatrics	78	2016–2017	99%	78%
Dang 2021^44^	Chemotherapy	26	Up to 2020	96%	92%
Abdel-Hamid 2023^43^	Premature ejaculation	15	Up to 2020	93%	93%
Pereira 2023^45^	Periodontology	127	2019 and 2020	89%	90.6%
Our study	Any biomedical topic	200	Up to 2023	73%	81%

In contrast, the majority of Cochrane SRs were of high methodological quality and low risk of bias. The findings that Cochrane SRs adhere to higher standards and are therefore at lower risk of bias than non-Cochrane reviews are generally supported in the literature.^49^^–^ ^51^ A portion of this trend might be attributable to Cochrane’s strict editorial standards (i.e., Methodological Expectations of Cochrane Intervention Reviews ^52^).

### 4.3 Specific concerns about under-reporting of items

Inadequate reporting of methods and results was seen in the majority of SRs (>50%) based on non-reported items in both ROBIS and AMSTAR-2 tools, making a full quality assessment not possible. Six AMSTAR-2 items we identified as inadequately reported in the majority of SRs (i.e., items 2, 3, 7, 10, 12, and 13) mirrored the findings of Guan and colleagues (2023) who also reported less than 50% ‘Yes/Partial Yes’ counts for items 2, 3, 7, 10, 12, and 13.^53^ Importantly, three of these under-reported items (i.e., items 2, 7, and 13) are critical items in AMSTAR-2 (namely items 2, 4, 7, 9, 11, 13, and 16). Eight items in the ROBIS tool were also under-reported by approximately less than half of the reviews (1.1, 1.5, 2.3, 2.4, 3.5, 4.2, 4.6, A). Default checks of these AMSTAR-2 and ROBIS elements in reviews may be warranted as the bare minimum when duplicate and independent assessment with these tools is not being done.

Recent work highlights critical shortcomings in the reporting of SRs without meta-analysis, which impacts quality assessments, including a lack of description of the methods used, a lack of transparent links between study-level data and the text reporting the synthesis and its conclusions, and inadequate reporting of the limitations of the synthesis.^54^ Another study of SRs without meta-analysis highlighted limitations related to determining clear eligibility criteria, inadequate search strategies, assessing, and addressing biases in primary studies.^55^

Based on these commonly missed AMSTAR-2 and ROBIS items, policy makers and clinicians may want to prioritise reviews with pre-published protocols, including data analysis plans. Noticeably, mere prospective registration of protocols on public platforms such as PROSPERO and their subsequent reporting in published SRs may not suffice the serious reporting-related shortcomings that we observed in the SRs. It is necessary to ensure that all protocol deviations are considered, recorded, and reported against any items the review authors designate as critical for their use case.

Furthermore, the SRs eligibility criteria and list of excluded studies should be scrutinised by assessors, as the unaccounted or inappropriate exclusion of studies based on eligibility criteria may result in missed studies, thus potentially impacting SR results and introducing bias. Finally, assessors should evaluate whether the risk of bias assessment of primary studies was sufficiently considered in light of the authors’ stated SRs results, particularly when reviews include NRSI or RCTs with variable risk of bias assessments.^24^ While both AMSTAR-2 and ROBIS ask the user to assess whether the SR authors presented their results in light of the bias in the primary studies, in our study, we found that these items are infrequently assessed. In our study, ROBIS item C showed that authors of SR frequently overstated the SR results based on imbalanced conclusions, highlighting only positive results. Thus, authors of SRs should discuss all SR outcomes, as well as the limitations of their methods and results, to provide balanced and unbiased conclusions.

### 4.4 Assessment time

A shorter median time was observed for AMSTAR-2 assessments than for ROBIS assessments (51 vs. 64 minutes), when these tools were applied first in order. Specifically, an average of 3.2 minutes per item was recorded for AMSTAR-2 when it was applied first, compared to approximately 2.7 minutes per item for ROBIS when it was applied first. When AMSTAR-2 was applied first, 69% of the assessments were completed in under 60 minutes, and 92% of the assessments were completed in under 90 minutes. In comparison, when ROBIS was applied first, 43% of the assessments were done in under 60 minutes, while 94% of the assessments were completed within 120 minutes. Both tools took less time to complete when applied second in order, compared to when applied first.

In our study, the median time to complete a ROBIS assessment was higher (excluding the time it took to read the SR) than the times presented in Perry et al.^35^ and Gates et al.,^33^ who included reading time. Pieper et al. reported that the time for using AMSTAR-2 was higher than ROBIS, with reading time included.^32^ Some of these differences may be explained by variation in time between assessors which was observed in our data similarly to Pieper’s study,^32^ but other factors such as rater experience, expertise in topic of the SR, decision rules used, learning effect when sequences are not altered, whether reading time was included, and the conceptual approach may also be relevant.

### 4.5 Agreement between assessors

Between senior and junior assessors, we found that agreement was high, with three-quarters of items showing more than 70% agreement, and one-quarter showing under 70%. The high agreement was likely due to (a) the clear and detailed guidance provided in addition to the ROBIS and AMSTAR-2 documentation, (b) the training on how to use these two tools, (c) piloting assessments with the supervision of a senior assessor, (d) having assessors provide quotes from the SR material to back up every item response, and (e) having random quality checks (and corrections) done by senior assessors.

Several empirical studies have compared AMSTAR-2 and ROBIS in terms of interrater reliability. Banzi et al.^30^ found similar interrater reliability for the AMSTAR v1 and ROBIS tools, with kappa values of 0.73 for AMSTAR v1 and 0.64 for ROBIS. In contrast, Pieper et al. found that in 30 SRs assessed first with AMSTAR-2 and then followed by ROBIS, the agreement between the four reviewers was fair (0.30 and 0.28, respectively).^32^ Gates et al. reported Gwet’s AC_1_, which are typically 0.10–0.20 higher than kappa values. Gates et al. applied the two instruments in three centres and reported Gwet’s AC (ROBIS range −0.21 to 0.56; AMSTAR-2 range 0.58–0.74), which translates to kappa values of approximately −0.45 to 0.32 for ROBIS and κ ≈ 0.49–0.65 for AMSTAR-2. Perry et al. found that both tools had similar AC_1_, with a median agreement of 0.61 for both.^35^ Because our own ratings are based on percentage agreement, our estimates are best viewed as an *upper bound* on what users can expect in routine multi-centre practice. Future methodological work should therefore prioritise blinded, duplicate ratings across multiple centres and report median and weighted kappa (or another chance-corrected statistic) so that tools can be compared on a common scale.

### 4.6 Implications for tool users

These findings have direct implications for the selection of appraisal tools in evidence synthesis practice. When used alone, AMSTAR-2 can be completed faster than ROBIS by assessors with different backgrounds and experience. A high percentage agreement between senior and junior assessors can be achieved on most items in both tools; however, the effects of training and piloting must be taken into consideration, as we did not evaluate them in a formal comparative fashion. Comprehensiveness of literature searches and the appropriateness of sources used should also be carefully considered by all users, as these were the only matched items that had less than 60% agreement.

Given that there is substantial but not full overlap between many items in the ROBIS and AMSTAR-2 tools, each tool may be more practical when specific elements unique to it need to be considered. ROBIS contains a fuller assessment of non-reporting and publication bias compared to AMSTAR-2. ROBIS also considers external validity and bias in the conclusions of a review, which is not present in the AMSTAR-2 tool. ROBIS can be used in scenarios when appropriateness of eligibility criteria, restrictions placed on these criteria, search strategy restrictions, relevance of results to study questions, collection and inclusion of all relevant studies in synthesis, minimisation of error in screening, reporting of pre-defined analyses and associated departures, and spin in the conclusions need to be evaluated and affect the use cases. AMSTAR-2 may be more feasible to use when methodological quality is of interest and in settings where faster assessment needs to be prioritised. AMSTAR-2 includes more features of quality, such as the reporting of conflict of interest, study funding, or a detailed list of excluded studies in SRs, that do not introduce a bias, and that are not included in ROBIS.

### 4.7 Limitations

Two major limitations affect our study results. First, we included a non-representative sample of SRs because of the need to include a greater percentage of high-quality reviews, including those published in the Cochrane Database of Systematic Reviews. Consequently, our sample is not representative of a random sample of SRs, as they would typically be of higher quality and higher risk of bias than what we found here. Second, only 83% of SRs were checked against our decision rules by senior assessors. Unchecked assessments that have not undergone quality checking may have missed quotes or the item may have been misinterpreted, which could potentially change the direction of the response rating. In addition, our item mapping analysis was not verified by the original authors of the two tools; thus, their opinions about how each item is categorised could differ from ours.

We also want to note that not all included SR were interventional, as some were epidemiological in nature. This may have affected the AMSTAR-2 rating, as this tool was not designed for these types of SRs, and our application may diverge from its proposed use. In addition, we did not state eligibility criteria around how a SR was defined; we simply included all SRs when the authors stated it as such in the title and abstract. Our rationale was that users of an artificial intelligence (AI) tool, *in development by our team*, to assess the quality of SRs, are unlikely to first vet the SR of their choosing against such eligibility criteria.

Furthermore, our calculation of percentage agreement does not account for agreement by chance. Also, percentage agreement often overestimates reliability, suggesting a higher level of agreement than is the case. Only 14 experienced assessors reported their assessment times, and a total of 61 individual SRs were timed. The results of the assessment times were not blinded to other assessors, which could have introduced bias towards reporting times that are closer to peers. We documented the aggregate completion time for each assessment employing the AMSTAR-2 or ROBIS tools. The completion time by item and domain was not recorded. The absence of item- or domain-specific timings hinders our capacity to discern which items or domains necessitate more time than others.

A larger sample with more assessors of different experience levels and backgrounds in evidence synthesis would have been preferable. However, assessors were recruited from a citizen science site (i.e., Cochrane Engage) with various backgrounds and different levels of experience, who tested the tools, which mimic real-world conditions where individuals with a range of expertise are involved in methodological quality and risk of bias appraisal. Our self-directed training, piloting, and development of decision rules and quality checks likely improved the standardisation of the assessments and contributed to the high percentage agreement we found. Future work should explore whether hybrid tools can balance comprehensiveness with efficiency.

### 4.8 Conclusions

In conclusion, we found that the majority of SRs assessed with the AMSTAR-2 and ROBIS tools were of low or critically low quality and had a high risk of bias, respectively. The majority of items in either tool overlapped fully or partially in content, with ROBIS containing a more comprehensive assessment of non-reporting and publication bias compared to AMSTAR-2. ROBIS also considers external validity and bias in the conclusions of a SR, which is not present in AMSTAR-2. ROBIS uniquely addressed the appropriateness of, and restrictions on eligibility criteria, reducing error in risk of bias assessments, the completeness of data extracted for analyses, the inclusion of all necessary studies for analyses, and adherence to a predefined analysis plan. AMSTAR-2 uniquely addressed the rationale for the inclusion of study designs, reporting on excluded studies with justification, sources of funding of primary studies, and reviewers’ conflicts of interest. However, the nine unique items in ROBIS, and the three unique items in AMSTAR-2, means that the two tools cannot be directly compared. This fact was also confirmed by our matched analysis of the overall judgments, showing that the 18 SRs where the ratings were in different directions were due to unique items in each of the tools.

The median time to complete the AMSTAR-2 was faster than that of ROBIS, with both taking under or over 1 hour to complete, respectively. The percentage agreement between raters was substantial, which is most likely due to our standardised training and piloting. In summary, AMSTAR-2 and ROBIS provide complementary rather than interchangeable assessments of systematic reviews. AMSTAR-2 may be preferable when efficiency is prioritized and methodological rigour is the focus, whereas ROBIS offers a deeper examination of potential biases and external validity. Given the widespread reliance on systematic reviews for policy and practice, selecting the appropriate appraisal tool remains crucial. Future research should explore strategies to integrate the strengths of both instruments while minimizing the burden on assessors.

## Supporting information

Lunny et al. supplementary material Lunny et al. supplementary material

## Data Availability

All data are available in the manuscript, supplementary material, the supplementary data files and in the Open Science Framework (https://osf.io/nbcta/). Further clarifications can be directed to the corresponding author.

## Supplementary material

To view supplementary material for this article, please visit http://doi.org/10.1017/rsm.2025.10032.

## References

[1] Lunny C , Whitelaw S , Reid EK , et al. Exploring decision-makers’ challenges and strategies when selecting multiple systematic reviews: insights for AI decision support tools in healthcare. BMJ Open. 2024;14(7):e084124. 10.1136/bmjopen-2024-084124 PMC11227798 38969371

[2] Page MJ , McKenzie JE , Bossuyt PM , et al. The PRISMA 2020 statement: an updated guideline for reporting systematic reviews. Syst Rev. 2021;10(1):1–11. 33781348 10.1186/s13643-021-01626-4 PMC8008539

[3] Higgins JP , Thomas J , Chandler J , et al. Cochrane Handbook for Systematic Reviews of Healthcare Interventions. John Wiley & Sons; 2019.

[4] Mhaskar R , Emmanuel P , Mishra S , Patel S , Naik E , Kumar A . Critical appraisal skills are essential to informed decision-making. Indian J Sex Transm Dis AIDS. 2009;30(2):112–119. 21938133 10.4103/2589-0557.62770 PMC3168054

[5] Page MJ , McKenzie JE , Kirkham J , et al. Bias due to selective inclusion and reporting of outcomes and analyses in systematic reviews of randomised trials of healthcare interventions. Cochrane Database Syst Rev. 2014;(10):MR000035. 25271098 10.1002/14651858.MR000035.pub2 PMC8191366

[6] Petticrew M . Why certain systematic reviews reach uncertain conclusions. BMJ. 2003;326(7392):756–758. 12676848 10.1136/bmj.326.7392.756 PMC1125658

[7] Sutton AJ , Duval SJ , Tweedie RL , Abrams KR , Jones DR . Empirical assessment of effect of publication bias on meta-analyses. BMJ. 2000;320(7249):1574–1577. 10845965 10.1136/bmj.320.7249.1574 PMC27401

[8] Boutron I , Page MJ , Higgins JP , Altman DG , Lundh A , Hróbjartsson A . Group CBM: considering bias and conflicts of interest among the included studies. In: Higgins JPT, Thomas J, Chandler J, Cumpston M, Li T, Page MJ, Welch VA, eds. Cochrane Handbook for Systematic Reviews of Interventions. John Wiley & Sons; 2019:177–204.

[9] Chiu K , Grundy Q , Bero L . ‘Spin’in published biomedical literature: a methodological systematic review. PLoS Biol. 2017;15(9):e2002173. 28892482 10.1371/journal.pbio.2002173 PMC5593172

[10] Demla S , Shinn E , Ottwell R , et al. Evaluation of ‘spin’ in the abstracts of systematic reviews and meta-analyses focused on cataract therapies. Am J Ophthalmol. 2021;228:47–57. 33823157 10.1016/j.ajo.2021.03.032

[11] McGrath TA , McInnes MD , van Es N , Leeflang MM , Korevaar DA , Bossuyt PM . Overinterpretation of research findings: evidence of ‘spin’ in systematic reviews of diagnostic accuracy studies. Clin Chem. 2017;63(8):1353–1362. 28606911 10.1373/clinchem.2017.271544

[12] Nascimento DP , Gonzalez GZ , Araujo AC , Moseley AM , Maher CG . Costa LOP: eight in every 10 abstracts of low back pain systematic reviews presented spin and inconsistencies with the full text: an analysis of 66 systematic reviews. J Orthop Sports Phys Ther. 2020;50(1):17–23. 31443622 10.2519/jospt.2020.8962

[13] Lunny C , Higgins JPT , White IR , et al. The RoB NMA tool: development of a tool to assess risk of bias (RoB) in a network meta-analysis (NMA). BMJ. 2025;388: e079839. 10.1136/bmj-2024-079839 PMC11915405 40101916

[14] Sterne JA , Savović J , Page MJ , et al. RoB 2: a revised tool for assessing risk of bias in randomised trials. BMJ. 2019;366: l4898. 10.1136/bmj.l4898 31462531

[15] Page MJ , McKenzie JE , Higgins JPT . Tools for assessing risk of reporting biases in studies and syntheses of studies: a systematic review. BMJ Open. 2018;8(3):e019703. 10.1136/bmjopen-2017-019703 PMC5857645 29540417

[16] O’Connor SR , Tully MA , Ryan B , Bradley JM , Baxter GD , McDonough SM . Failure of a numerical quality assessment scale to identify potential risk of bias in a systematic review: a comparison study. BMC Res Notes 2015; 8:1–7. 26048813 10.1186/s13104-015-1181-1 PMC4467625

[17] Sanderson S , Tatt ID , Higgins JP . Tools for assessing quality and susceptibility to bias in observational studies in epidemiology: a systematic review and annotated bibliography. Int J Epidemiol. 2007;36(3):666–676. 17470488 10.1093/ije/dym018

[18] Long HA , French DP , Brooks JM . Optimising the value of the critical appraisal skills programme (CASP) tool for quality appraisal in qualitative evidence synthesis. Res Meth Med Health Sci. 2020;1(1):31–42.

[19] Boutron I , Dutton S , Ravaud P , Altman DG . Reporting and interpretation of randomized controlled trials with statistically nonsignificant results for primary outcomes. JAMA. 2010;303(20):2058–2064. 20501928 10.1001/jama.2010.651

[20] Jankowski S , Boutron I , Clarke M . Influence of the statistical significance of results and spin on readers’ interpretation of the results in an abstract for a hypothetical clinical trial: a randomised trial. BMJ Open. 2022;12(4):e056503. 10.1136/bmjopen-2021-056503 PMC8996040 35396295

[21] Green LW , Glasgow RE . Evaluating the relevance, generalization, and applicability of research: issues in external validation and translation methodology. Eval Health Prof. 2006;29(1):126–153. 16510882 10.1177/0163278705284445

[22] Murad MH , Katabi A , Benkhadra R , Montori VM . External validity, generalisability, applicability and directness: a brief primer. BMJ Evid Based Med. 2018;23(1):17–19. 10.1136/ebmed-2017-110800 29367319

[23] Whiting P , Savović J , Higgins JP , et al. ROBIS: a new tool to assess risk of bias in systematic reviews was developed. J Clin Epidemiol. 2016;69:225–234. 26092286 10.1016/j.jclinepi.2015.06.005 PMC4687950

[24] Shea BJ , Reeves BC , Wells G , et al. AMSTAR 2: a critical appraisal tool for systematic reviews that include randomised or non-randomised studies of healthcare interventions, or both. BMJ. 2017;358: j4008. 10.1136/bmj.j4008 PMC5833365 28935701

[25] Bojcic R , Todoric M , Puljak L . Adopting AMSTAR 2 critical appraisal tool for systematic reviews: speed of the tool uptake and barriers for its adoption. BMC Med Res Methodol. 2022;22(1):104. 35399051 10.1186/s12874-022-01592-y PMC8996416

[26] Burda BU , Holmer HK , Norris SL . Limitations of a measurement tool to assess systematic reviews (AMSTAR) and suggestions for improvement. Syst Rev. 2016;5(1):1–10. 27072548 10.1186/s13643-016-0237-1 PMC4830078

[27] Faggion CM . Critical appraisal of AMSTAR: challenges, limitations, and potential solutions from the perspective of an assessor. BMC Med Res Methodol. 2015;15(1):1–5. 26268372 10.1186/s12874-015-0062-6 PMC4535290

[28] Wegewitz U , Weikert B , Fishta A , Jacobs A , Pieper D . Resuming the discussion of AMSTAR: what can (should) be made better? BMC Med Res Methodol. 2016;16(1):1–8. 27566440 10.1186/s12874-016-0183-6 PMC5002206

[29] Whiting P , Savović J , Higgins J , et al. Guidance on How to Use ROBIS. Bristol University; 2015. https://www.bristol.ac.uk/media-library/sites/social-community-medicine/robis/ROBIS%20Report%204_9.pdf.

[30] Banzi R , Cinquini M , Gonzalez-Lorenzo M , Pecoraro V , Capobussi M , Minozzi S . Quality assessment versus risk of bias in systematic reviews: AMSTAR and ROBIS had similar reliability but differed in their construct and applicability. J Clin Epidemiol. 2018;99:24–32. 29526556 10.1016/j.jclinepi.2018.02.024

[31] Buehn S , Mathes T , Prengel P , et al. The risk of bias in systematic reviews tool showed fair reliability and good construct validity. J Clin Epidemiol. 2017;91:121–128. 28694122 10.1016/j.jclinepi.2017.06.019

[32] Pieper D , Puljak L , González-Lorenzo M , Minozzi S . Minor differences were found between AMSTAR 2 and ROBIS in the assessment of systematic reviews including both randomized and nonrandomized studies. J Clin Epidemiol. 2019;108:26–33. 30543911 10.1016/j.jclinepi.2018.12.004

[33] Gates M , Gates A , Duarte G , et al. Quality and risk of bias appraisals of systematic reviews are inconsistent across reviewers and centers. J Clin Epidemiol. 2020;125:9–15. 32416337 10.1016/j.jclinepi.2020.04.026

[34] Lorenz RC , Matthias K , Pieper D , et al. A psychometric study found AMSTAR 2 to be a valid and moderately reliable appraisal tool. J Clin Epidemiol. 2019;114:133–140. 31152864 10.1016/j.jclinepi.2019.05.028

[35] Perry R , Whitmarsh A , Leach V , Davies P . A comparison of two assessment tools used in overviews of systematic reviews: ROBIS versus AMSTAR-2. Syst Rev. 2021;10(1):273. 34696810 10.1186/s13643-021-01819-x PMC8543959

[36] Shea BJ , Bouter LM , Peterson J , et al. External validation of a measurement tool to assess systematic reviews (AMSTAR). PLoS One. 2007;2(12):e1350. 18159233 10.1371/journal.pone.0001350 PMC2131785

[37] Von Elm E , Altman D , Egger M , Pocock S , Gøtzsche P , Vandenbroucke J . STROBE checklist: cohort, case-control, and cross-sectional studies (combined). Lancet. 2007;370(9596):1453–1457. 18064739 10.1016/S0140-6736(07)61602-X

[38] Smires S , Afach S , Mazaud C , et al. Quality and reporting completeness of systematic reviews and meta-analyses in dermatology. J Invest Dermatol. 2021;141(1):64–71. 32603750 10.1016/j.jid.2020.05.109

[39] Schünemann HJ , Mustafa RA , Brozek J , et al. GRADE guidelines: 21 part 1. Study design, risk of bias, and indirectness in rating the certainty across a body of evidence for test accuracy. J Clin Epidemiol. 2020;122:129–141. 32060007 10.1016/j.jclinepi.2019.12.020

[40] Yaffe J , Montgomery P , Hopewell S , Shepard LD . Empty reviews: a description and consideration of Cochrane systematic reviews with no included studies. PLoS One. 2012;7(5):e36626. 22574201 10.1371/journal.pone.0036626 PMC3344923

[41] Pollock M , Fernandes RM , Becker LA , Pieper D , Hartling L . Chapter V: overviews of reviews. In: Higgins JPT, Thomas J, Chandler J, Cumpston M, Li T, Page MJ, Welch VA, eds. Cochrane Handbook for Systematic Reviews of Interventions Version. John Wiley & Sons; 2020:6.

[42] Aromataris E , Fernandez RS , Godfrey C , Holly C , Khalil H , Tungpunkom P . Chapter 10: umbrella reviews. In: Aromataris E , Munn Z , eds. JBI Manual for Evidence Synthesis. JBI; 2014. https://jbi-global-wiki.refined.site/space/MANUAL.

[43] Abdel-Hamid IA , Abo-Aly M , Mostafa T . Phosphodiesterase type 5 inhibitors and premature ejaculation: an overview of systematic reviews/meta-analyses using the AMSTAR 2, ROBIS, and GRADE tools. Sex Med Rev. 2023;11(1):23–51.

[44] Dang A , Chidirala S , Veeranki P , Vallish B . A critical overview of systematic reviews of chemotherapy for advanced and locally advanced pancreatic cancer using both AMSTAR2 and ROBIS as quality assessment tools. Rev Recent Clin Trials. 2021;16(2):180–192. 32875987 10.2174/1574887115666200902111510

[45] Pereira AG , Martins CC , Campos JR , et al. Critical appraisal of systematic reviews of intervention studies in periodontology using AMSTAR 2 and ROBIS tools. J Clin Exp Dent. 2023;15(8):e678–e694. 37674600 10.4317/jced.60197 PMC10478201

[46] Storman M , Storman D , Jasinska KW , Swierz MJ , Bala MM . The quality of systematic reviews/meta‐analyses published in the field of bariatrics: a cross‐sectional systematic survey using AMSTAR 2 and ROBIS. Obes Rev. 2020;21(5):e12994. 31997545 10.1111/obr.12994

[47] Zajac JF , Storman D , Swierz MJ , et al. Are systematic reviews addressing nutrition for cancer prevention trustworthy? A systematic survey of quality and risk of bias. Nutr Rev. 2022;80(6):1558–1567. 34921318 10.1093/nutrit/nuab093 PMC9086792

[48] Kolaski K , Romeiser Logan L , Goss KD , Butler C . Quality appraisal of systematic reviews of interventions for children with cerebral palsy reveals critically low confidence. Dev Med Child Neurol. 2021;63(11):1316–1326. 34091900 10.1111/dmcn.14949

[49] Collier A , Heilig L , Schilling L , Williams H , Dellavalle RP . Cochrane skin group systematic reviews are more methodologically rigorous than other systematic reviews in dermatology. Br J Dermatol. 2006;155(6):1230–1235. 17107394 10.1111/j.1365-2133.2006.07496.x

[50] Moseley AM , Elkins MR , Herbert RD , Maher CG , Sherrington C . Cochrane reviews used more rigorous methods than non-Cochrane reviews: survey of systematic reviews in physiotherapy. J Clin Epidemiol. 2009;62(10):1021–1030. 19282144 10.1016/j.jclinepi.2008.09.018

[51] Tricco AC , Tetzlaff J , Pham B , Brehaut J , Moher D . Non-cochrane vs. cochrane reviews were twice as likely to have positive conclusion statements: cross-sectional study. J Clin Epidemiol. 2009;62(4):380–386.e381. 19128940 10.1016/j.jclinepi.2008.08.008

[52] Swierz MJ , Storman D , Zajac J , et al. Similarities, reliability and gaps in assessing the quality of conduct of systematic reviews using AMSTAR-2 and ROBIS: systematic survey of nutrition reviews. BMC Med Res Methodol. 2021;21(1):261. 34837960 10.1186/s12874-021-01457-w PMC8627612

[53] Guan X , Lao Y , Wang J , et al. The methodological quality assessment of systematic reviews/meta-analyses of chronic prostatitis/chronic pelvic pain syndrome using AMSTAR2. BMC Med Res Methodol. 2023;23(1):281. 38012566 10.1186/s12874-023-02095-0 PMC10680214

[54] Campbell M , Katikireddi SV , Sowden A , Thomson H . Lack of transparency in reporting narrative synthesis of quantitative data: a methodological assessment of systematic reviews. J Clin Epidemiol. 2019;105:1–9. 30196129 10.1016/j.jclinepi.2018.08.019 PMC6327109

[55] Cumpston MS , Brennan SE , Ryan R , McKenzie JE . Synthesis methods other than meta-analysis were commonly used but seldom specified: survey of systematic reviews. J Clin Epidemiol. 2023;156:42–52. 36758885 10.1016/j.jclinepi.2023.02.003

