# Supplementary material for: Exploring the methodological quality and risk of bias in 200 systematic reviews: A comparative study of ROBIS and AMSTAR-2 tools
Source: Res Synth Methods. 2025 Oct 27;17(1):63–92. doi: 10.1017/rsm.2025.10032 (PMC12823211; doi:10.1017/rsm.2025.10032)
Supplement: Lunny et al. supplementary material [file S175928792510032Xsup001.docx]

**Appendices for the manuscript “Exploring the methodological quality and risk of bias in 200 systematic reviews: A comparative study of ROBIS and AMSTAR-2 tools” by Lunny et al. (2025)**

Appendix A: Strengthening of Reporting of Observational Studies in Epidemiology (STROBE) for cross-sectional studies checklist

Appendix B: Cochrane Database of Systematic Reviews search strategy

Appendix C: Decision rules for how to interpret the ROBIS and AMSTAR-2 questions

Appendix D: Data elements extracted

Appendix E: Comparison of overall judgments and direction of ratings

Appendix F: Comparison of ROBIS and AMSTAR-2 item ratings

**Appendix A: Strengthening of Reporting of Observational Studies in Epidemiology (STROBE) for cross-sectional studies checklist**

| **Item** | **#** | **Recommendation** | **Page  No.** |
| --- | --- | --- | --- |
| Title and abstract | 1 | (*a*) Indicate the study’s design with a commonly used term in the title or the abstract | 1 |
|  |  | (*b*) Provide in the abstract an informative and balanced summary of what was done and what was found | 4 |
| Introduction | | | |
| Background/rationale | 2 | Explain the scientific background and rationale for the investigation being reported | 7-8 |
| Objectives | 3 | State specific objectives, including any prespecified hypotheses | 8 |
| Methods | | | |
| Study design | 4 | Present key elements of study design early in the paper | 8 |
| Setting | 5 | Describe the setting, locations, and relevant dates and data collection | 8-9 |
| Population (SRs) | 6 | *Cross-sectional study*—Give the eligibility criteria, and the sources and methods of selection of SRs | 8 |
|  |  |  | 8-9 |
| Variables | 7 | Clearly define all outcomes | 8-10 |
| Data sources/ measurement | 8* | For each variable of interest, give sources of data and details of methods of assessment (measurement). | 9 |
| Bias | 9 | Describe any efforts to address potential sources of bias | 8-9 |
| Study size | 10 | Explain how the study size was arrived at | N/A |
| Quantitative variables | 11 | Explain how quantitative variables were handled in the analyses. If applicable, describe the stratification chosen and why | 10 |
| Statistical methods | 12 | (*a*) Describe all statistical methods, including those used to control for confounding | 10-11 |
|  |  | (*b*) Describe any methods used to examine subgroups and interactions | N/A |
|  |  | (*c*) Explain how missing data were addressed | 10-11 |
|  |  | (*d*) *Cross-sectional study*—If applicable, describe analytical methods taking account of sampling strategy | 10-11 |
|  |  | (*e*) Describe any sensitivity analyses | N/A |
| RESULTS |  |  |  |
| Population | 13* | (a) Report numbers of SRs—eg numbers included in the study, and analysed | 11 |
|  |  | (b) Consider use of a flow diagram | N/A |
| Descriptive data | 14* | (a) Give characteristics of SRs (eg conditions, types) | Table 2 |
|  |  | (b) Indicate number of SRs with missing data for each variable of interest | Table 2 |
| Outcome data | 15* | *Cross-sectional study—*Report numbers of outcome events or summary measures | Table 2 |
| Main results | 16 | (*a*) Give unadjusted estimates and, if applicable, confounder-adjusted estimates and their precision (eg, 95% confidence interval). | N/A |
|  |  | (*b*) Report category boundaries when continuous variables were categorized | Table 2 |
|  |  | (*c*) If relevant, consider translating estimates of relative risk into absolute risk for a meaningful time period | N/A |
| Other analyses | 17 | Report other analyses done—eg analyses of subgroups and interactions, and sensitivity analyses | N/A |
| DISCUSSION |  |  |  |
| Key results | 18 | Summarise key results with reference to study objectives | 23 |
| Limitations | 19 | Discuss limitations of the study, taking into account sources of potential bias or imprecision. Discuss both direction and magnitude of any potential bias | 25-26 |
| Interpretation | 20 | Give a cautious overall interpretation of results considering objectives, limitations, multiplicity of analyses, results from similar studies, and other relevant evidence | 26 |
| Generalisability | 21 | Discuss the generalisability (external validity) of the study results | 25 |
| Other information |  |  |  |
| Funding | 22 | Give the source of funding and the role of the funders for the present study and, if applicable, for the original study on which the present article is based | 28-29 |

***Abbreviation:*** *N/A – not applicable.*

**Appendix B: Cochrane Database of Systematic Reviews search strategy**

A total of 36 Cochrane systematic reviews were retrieved from the Cochrane Database of Systematic Reviews (date September 14, 2023).

The totality of the Cochrane SRs at that time was 9400. These were randomly sorted using Excel’s Rand function and categorised into the following 50 topics as reported from the Cochrane library’s taxonomy (**Box A**): acute respiratory infections; airways; anaesthesia; back and neck; Bone, Joint and Muscle Trauma; breast cancer; childhood cancer; colorecta; mental disorders; Schizophrenia; Consumers and Communication; Cystic Fibrosis and Genetic Disorders; Dementia and Cognitive Improvement; Developmental, Psychosocial and Learning Problems; ; Drugs and Alcohol; Effective Practice and Organisation of Care; Emergency and Critical Care; ENT; Epilepsy; Eyes and Vision; Fertility Regulation; gut; Gynaecology and Fertility; Gynaecological, Neuro-oncology and Orphan Cancer; Haematology; Heart; Stroke;; Hepato-Biliary; HIV/AIDS; Hypertension; Incontinence; Infectious Diseases; Injuries; Kidney and Transplant; Lung Cancer; Metabolic and Endocrine Disorders; Movement Disorders; Multiple Sclerosis and Rare Diseases of the CNS; ; Neonatal; Neuromuscular; Pain; Palliative and Supportive Care; Pregnancy and Childbirth; Public Health; Sexually Transmitted Infections; Tobacco Addiction; Urology; Work, and Wounds.

Ten randomly selected SRs per topic were retained. These 500 SRs were given ID numbers and added to our larger WISEST AI dataset for assessment. Of these 500 SRs, 36 Cochrane SRs were assessed and retained for inclusion in our methods study.

| **Box A:** **Cochrane library Taxonomy** | |
| --- | --- |
| A | I |
| Acute Respiratory infections  Airways  Anesthesia | Incontinence  Infectious Diseases  Injuries |
| B | K |
| Back & neck  Bone, joint & Muscle Trauma  Breast Cancer | Kidney and Transplant  Lung Cancer |
| C | M |
| Childhood cancer  Colorectal  Consumers and Communication  Cystic Fibrosis and Genetic Disorders | Metabolic and Endocrine Disorders  Movement Disorders  Multiple Sclerosis and Rare Disease of the CNS |
| Dementia and Cognitive Improvement  Developmental, Psychosocial and Learning Problems Drugs and Alcohol | Neonatal  Neuromuscular |
| E | P |
| Effective practice and Organization of Care  Emergency and Critical Care  ENT  Epilepsy  Eyes and Vision | Pain  Palliative and Supportive Care  Pregnancy and Childbirth  Public Health |
| F | S |
| Fertility Regulation | Sexual Transmitted Infections |
| G | T |
| Gut  Gynaecology & Fertility  Gynaecological, Neuro-oncology and Orphan Cancer | Tobacco addiction  Urology |
| Haematology  Heart  Hepato-Biliary  HIV/AIDS  Hypertension | Work  Wounds |

We applied the following eligibility criteria to the retrieved SRs:

- SRs with and without meta-analysis of interventions and epidemiology
- SRs with pairwise or no meta-analysis,
- SRs containing any primary study designs (e.g. RCTs and non-RCTs);
- SRs entitled systematic review in the title or abstract

We excluded:

- Any SRs not of interventions or epidemiology (e.g., diagnostic accuracy, methodology)
- SRs with network meta-analysis, individual patient data [IPD] meta-analysis

**Appendix C: Decision rules for how to interpret the ROBIS and AMSTAR-2 questions**

| **ROBIS** | **AMSTAR-2** | **Decision rule** |
| --- | --- | --- |
| 1.1 Did the review adhere to pre-defined objectives and eligibility criteria? | 2.Did the report of the review contain an explicit statement that the review methods were established prior to the conduct of the review and did the report justify any significant deviations from the protocol? | A. Was a protocol, PROSPERO registry, or registered report written by the authors, and located by the assessor? If yes, then: B. Compare the objectives and eligibility criteria in the protocol with those in the manuscript • If any differences found, then put "No" • If no differences found then "Yes" • If differences found, but they describe these 'deviations' in the manuscript of the review, then put "Yes" • If no protocol found then put "No "  For AMSTAR-2, no protocol found then put "No" |
| 1.2 Were the eligibility criteria appropriate for the review question? |  | Match question/study design to PICO eligibility criteria. A. Is the PICO appropriate to the research question? If no, use another pneumonic like PCC [population, concept, context]. |
| 1.3 Were eligibility criteria unambiguous? | 1.Did the research questions and inclusion criteria for the review include the components of PICO? | A. Is the PICO components used to develop the eligibility criteria? B. If Population and Interventions are named/ described, then put "Yes". Can be in the abstract, background, or methods section |
|  | 3. Did the review authors explain their selection of the study designs for inclusion in the review? | Rationale for why they include certain study designs (e.g. RCTs. Cohort studies).  For example for RCTs, they might say that they want to prove causation for the efficacy of a drug treatment. For observational studies, they may say that it is unethical to conduct RCTs in this field of research, etc. |
| 1.4 Were all restrictions in eligibility criteria based on study characteristics appropriate (e.g. date, sample size, study quality, outcomes measured)? If yes, indicate which study characteristic was an inclusion/exclusion criterion |  | A. Were studies excluded SPECIFICALLY because of sample size, study quality, outcomes, date, or other study characteristic (e.g. missing results data)? B. If Yes, then state which characteristic above was excluded, and whether it was appropriate to include/exclude them based on these characteristics (i.e. did the authors have a sound rationale for why this was and eligibility criteria?) C. If there is nothing in the manuscript about this, or in the supplements/appendices (check the search strategy), then put "No Information"  As a note: Many papers are high quality but do not report any restrictions for their eligibility criteria nor their search strategy. In the domain judgment, we do not consider this item unless it is a Yes or a No (i.e. No Information is ignored).  Example: Often reviews will say they exclude studies because they did not contain the outcomes they were interested in, or the outcomes were not in the proper format. If they excluded the studies for this reason, this is NOT appropriate and would be a No response to this item. Authors should include all studies if it meets their study design, population and intervention eligibility criteria. They would then contact the authors of the study to ask about the outcome they are interested in. If there is no reply, then the study can be categorised as “awaiting assessment” and described qualitatively by review authors. |
| 1.5 Were any restrictions in eligibility criteria based on sources of info appropriate (e.g. publication status or format, language, availability of data)? |  | A. Were eligible studies excluded because of language, or whether they were published (only in peer-review journals) or not (i.e. unpublished reports = conference abstracts, preprints, WHO reports, etc), availability of data? --If English language only were searched and included, then put "No" B. If yes, then was it appropriate to exclude them based on these characteristics? Put No if only English language was searched or eligibility criteria stated English studies only were included.  C. If there is nothing in the manuscript about this, or in the supplements/appendices (check the search strategy), then put "No Information"  If "No information" is found than indicate it as such here. as many papers are high quality but do not have any restrictions in their search. In the domain judgment, we do not consider this item unless it is a Yes or a No (i.e. No Information is ignored). |
| ROBIS Concerns regarding specification of eligibility criteria (low, high, unclear) |  | AUTOMATED DO NOT TOUCH – this is an excel macro which is automated  ROBIS:  Low risk: If: (i) item 1.1. is 'No info" or Yes/Probably Yes, (ii) items 1.2 and 1.3 are Yes/Probably Yes, AND (iii) items 1.4 and 1.5 are 'Yes' or 'No Information' High risk: If item 1.1, 1.2, 1.3, 1.4 and 1.5 are 'No'/'Probably No' |
| 2.1 Did the search include an appropriate range of databases/ electronic sources for published and unpublished reports? | 4. Did the review authors use a comprehensive literature search strategy? | To say Yes to this item: • Searched 2 or more databases of published ‘peer reviewed’ studies/articles AND searched 'grey' literature' defined as Information produced on all levels of government, academics, business and industry in electronic and print formats not controlled by commercial publishing i.e. where publishing is not the primary activity of the producing body. • Grey literature is anything NOT published in peer reviewed journals: Google, trial registries (e.g. clinicaltrials.gov), conference abstracts, dissertations, Preprints, PROSPERO, websites from government agencies, research institutes, organizations or companies, or associations  The Cochrane Centre Register of Controlled Trials DOES include grey literature so “Yes” if it is searched. |
| 2.2 Were methods additional to database searching used to identify relevant reports? |  | To say Yes to this item, they may have: • Searched the reference lists/bibliographies of included studies; OR • Forward citation searching; OR • Consulted content experts in the field • Other (e.g. archival data searched)  Note: Forward Citation Searching is a search to find all of the articles that cite back to a specific article. This search looks forward in time to see how this article contributed to the scholarly conversation. |
| 2.3 Were the terms and structure of the search strategy likely to retrieve as many eligible studies as possible? |  | To say Yes to this item: • Provided keywords and/or search strategy algorithm in the main manuscript or an appendix, with how they combined the terms AND  • the search would have retrieved all the studies in existence (i.e. is the search comprehensive enough, and keywords correctly linked to be able to find all the studies in existence on this topic?)  Note: The search should also be reproducible |
| 2.4 Were search strategy restrictions based on date, publication format, or language appropriate? |  | In the search strategy section of the manuscript, or in the Appendix with the full search reported: • Were restrictions on their search appropriate, such as date range, publication format, or language AND included an adequate rationale?  If "No information" is found than indicate it as such here. as many papers are high quality but do not have any restrictions in their search. In the domain judgment, we do not consider this item unless it is a Yes or a No (i.e. No Information is ignored). |
| 2.5 Were efforts made to minimise error in selection of studies? | 5.Did the review authors perform study selection in duplicate? | To say Yes to this item, the authors MUST have looked at citations of abstracts and full text: • Two reviewers screened the citations retrieved from the search, OR • One author screened studies, and a second author checked the "excluded citations"  • Two reviewers selected a sample of eligible studies and achieved good agreement (at least 80 percent), with the remainder selected by one reviewer  To say "No": • One author extracted data • One reviewer extracted data and a second randomly quality checked their data extraction  Item 2.5 (and 3.1) if there is nothing reported then put "No" instead of "No Information" |
| ROBIS Concerns regarding methods used to identify and/or select studies (low, high, unclear) |  | Automated so do not change - AUTOMATED DO NOT TOUCH – this is an excel macro which is automated  ROBIS:  Low concerns: If items 2.1, 2.2, 2.3 and 2.5 are "Yes", and 2.4 is "No info" or "Yes"  High concerns: If items 2.1, 2.2, 2.3 and 2.5 are "No", and 2.4 is a "No" |
| 3.1 Were efforts made to minimise error in data collection? | 6.Did the review authors perform data extraction in duplicate? | To say "Yes/Probably Yes" to this item: • Two reviewers independently extracted data and compared their extractions OR • Two reviewers extracted data from a sample of eligible studies and achieved good agreement (at least 80%), with the remainder extracted by one reviewer  To say "No": • One author extracted data • One author extracted, and a second author checked it on a random sample  If there is No information, here a No info will = No. So please put No. This item will not be used in the domain level judgment. |
|  | 7.Did the review authors provide a list of excluded studies and justify the exclusions? | To say Yes/Partial to this item: • Provided a list of all potentially relevant studies (in an appendix/supplementary file) that were read in full-text form but excluded from the review with reasons  No information: • If nothing mentioned in the SR, then put no information |
| 3.2 Were sufficient study characteristics considered for both review authors and readers to be able to interpret the results? | 8.Did the review authors describe the included studies in adequate detail? | To respond "Yes/Probably Yes" (usually in a table of characteristics or described in results section): • Described population  • Described intervention If there is No information, here a “No info” will = No. So please put No. This item will not be used in the domain level judgment.  Please include the title of the table and possible the table headers as a quote |
| 3.3 Were all relevant study results collected for use in the synthesis? |  | “Yes/Probably Yes”: Data are in the review that can be used to replicate the analysis. So for a meta-analysis, you would need the sample sizes of the trials, the number of "events" for the treatment and the control groups, and characteristics of the population/interventions so you could do subgroup analyses on these charcteristics that might be effect modifiers/confounders. Look in the Table of Characteristics, or the forest plots, or supplementary files if not readily apparent. If there is No information, here a No info will = No. So please put No. This item will not be used in the domain level judgment.  Yes description ROBIS: In the methods section authors described how results data that were not reported in the format required for synthesis were obtained e.g. by estimating/ transforming from reported data or by contacting authors for additional info. If these data are not reported in the methods section, it may be possible to work out what data were extracted from results tables, graphical summaries (e.g. forest plots) or data reported in the text. It may also be necessary to access additional resources such as web appendices or the review protocol if available. For example, (1) in a Diagnostic Test Accuracy review, 2x2 data were extracted from each study which is sufficient to calculate all measures of diagnostic accuracy and associated confidence intervals and to perform a meta-analysis.(2) in an intervention review, dichotomous data were extracted as number of events and total number of patients in each treatment arm; these were used to calculate relative risks. This was appropriate for use in the synthesis. |
| 3.4 Was risk of bias (or methodological quality) formally assessed using appropriate criteria? | 9.Did the review authors use a satisfactory technique for assessing the risk of bias (RoB) in individual studies that were included in the review? | To respond "Yes": • If the SR publication date was prior to 2008 and the authors assessed RCTs: used the Cochrane RoB 1.0 or 2.0 tool (note: Downs and Black tool (1998) to assess RCTs is not a valid tool as it does not include allocation concealment)  ---If published after 2008, a variety of tools/criteria may have been used. Consult with Carole if unsure. • If Non-RCTs: ROBINS-I, Newcastle Ottawa Scale, CASP, JBI, and other tools TBA  3.4 and 4.6 are "NO" if there is no information provided as the authors should have done this.  NOT appropriate, and a "NO" if theses tools are used: Jadad scoring system to appraise RCTs, the Downs and Black’s checklist for non-RCTs. They are old tools and are missing key domains of bias (e.g. Jadad is missing allocation concealment). |
| 3.5 Were efforts made to minimise error in risk of bias assessment? |  | To say "Yes/Probably Yes" to this item: • Two reviewers independently assessed risk of bias of the primary studies and compared their assessments OR • Two reviewers assessed risk of bias of the primary studies from a sample of eligible studies and achieved good agreement (at least 80%), with the remainder extracted by one reviewer To say "No": • One author extracted data • One author extracted, and a second author checked it on a random sample If there is No information, here a 'No information' will = No. So please put No. |
| ROBIS Concerns regarding methods used to collect data and appraise studies (low, high, unclear) |  | Automated so do not change  ROBIS:  Low risk: If items 3.1, 3.2 and 3.4 are "Yes/Probably Yes" (item 3.3 not taken into consideration) High risk: If items 3.1, 3.2 and 3.4 are "No" or "No Info" (item 3.3 not taken into consideration) |
| 4.1 Did the synthesis include all studies that it should? |  | Respond "No/Probably No" if you examine the numbers of included studies (e.g. from a flowchart) and the numbers of synthesized data from the studies (e.g. in forest plots or tables) and a mismatch is found; AND you suspect: (i) authors failed to collect or process the data available, or  (ii) authors purposefully excluded the results For example, reviewers extract data for an outcome (e.g. mortality) but report no results for it. The important distinction is whether the result was likely to have been suppressed because of the finding (e.g. lack of statistical significance) or simply not reported (e.g. for practical reasons such as a short paper length). Another example would be inappropriately excluding studies deemed to be driving a large between-studies heterogeneity based on statistical considerations alone (although this may be reasonable as a sensitivity analysis). Respond "Yes/Probably Yes" If you think they included all results from the studies in an analysis, or mentioned why they did not do this. They must state why a paper was not included if the outcomes are not discussed. |
| 4.2 Were all pre-defined analyses reported or departures explained? |  | Respond "Yes/Probably Yes" if there is an indication that predefined analyses were followed from a protocol; AND • You check the protocol to see if the analyses listed there are in the published manuscript  • All analyses listed in the protocol are included in the manuscript or supplementary files, or a rationale is provided why they are not included. Respond "No/Probably No": • If there was no protocol identified; AND there is suspicion of foul play - authors may have deleted/omitted a common analysis or outcome. Look at the clinical topic and think about what outcomes/analyses should have been examined. If a common outcome is not present, it may indicate foul play. • If an analysis is missing from the published manuscript that was pre-specified in the protocol, and there is no mention of why it is omitted  Respond "No Information": • There was no protocol and you do not suspect foul play |
| 4.3 Was the synthesis appropriate given the nature and similarity in the research questions, study designs and outcomes across included studies? | 11.If meta-analysis was performed did the review authors use appropriate methods for statistical combination of results? | If meta-analysis was conducted, then respond "Yes" if: • Random effects or fixed effects model for the meta-analysis was done AND • they conducted separate meta-analyses for RCTs and non-randomized studies when both were included in the review AND • The meta-analysis did not mix up different types of interventions/treatments (e.g. all types of chinese medicine treatment, such as acupuncture, herbs, cupping) BUT If no meta-analysis was done, then answer "Yes" if  • Some description of how the studies would be summarised (in section entitled methods/data analysis). If they said they are doing a descriptive analysis, narrative review, narrative summary, etc. OR • If they said that the studies were too different/varied to combine in a meta-analysis |
| 4.4 Was between-study variation (heterogeneity) minimal or addressed in the synthesis? | 14.Did the review authors provide a satisfactory explanation for, and discussion of, any heterogeneity observed in the results of the review? | If meta-analysis was conducted, respond "Yes" if: • High heterogeneity was present (I2=50%+) AND authors investigated the causes with subgroup, sensitivity analyses, or meta-regression analyses (authors attempt to figure out where the heterogeneity came from) • A narrative synthesis was conducted, respond “Yes”, since heterogeneity was addressed by not combining. • No clinical or statistical heterogeneity was present (I2= 49% or under) AND the PICO of the included studies are similar  If no meta-analysis was conducted, then respond "No meta-analysis" |
| 4.5 Were the findings robust, e.g. as demonstrated through funnel plot or sensitivity analyses? | 15.If they performed quantitative synthesis did the review authors carry out an adequate investigation of publication bias (small study bias) and discuss its likely impact on the results of the review? | If no meta-analysis was conducted, then respond "No meta-analysis"  A. Funnel Plots: 1. If meta-analysis conducted, and 10+ studies included, respond 'Yes/Probably Yes' when these analyses indicated NO publication bias: • Funnel plot, Egger's test, Peter's Test, Harbord's test, Trim-and-fill method, rank correlation test (e.g. Begg's test), Caliper test, or Copas and Jackson's selection model was conducted  OR  • There is a quote recognizing the publication bias (e.g. “The presence of publication bias was not examined in this systematic review because there were insufficient trials to allow meaningful formal assessment using funnel plots”) 2. When 9 studies or less were included: respond "Not applicable: NA" and do not consider this item in the domain-level judgment 3. Respond "No/Probably No" when 10+ studies included and: • Did not explore publication bias through any of the listed methods (Note do not put "No Information" OR • Publication bias was present as indicated by these tests  Note: Publication bias refers to the selective publication of research findings based on their statistical significance, which can result in a distorted or incomplete picture of the true state of knowledge. It occurs when studies with statistically significant results are more likely to be published than those with non-significant results or negative findings. This can lead to an overestimation of the effect size of a particular intervention or treatment. Small study bias, on the other hand, refers to the tendency for small studies to have more extreme or variable results than larger studies. This can occur due to various factors, such as sampling variability, measurement error, or publication bias. Small study bias can lead to inaccurate or exaggerated effect sizes, as well as difficulties in replicating findings in larger studies. While publication bias can contribute to small study bias, they are not the same thing, and each can occur independently of the other. Funnel plot: A funnel plot is a scatter plot that can be used to visualize the relationship between the effect size and sample size of each study included in the meta-analysis. If there is no small study effect, the plot should resemble a symmetrical funnel. Asymmetry in the plot can indicate the presence of small study effects or publication bias.  B. Sensitivity analyses: Sensitivity analyses compare how different methods affect the effect estimates (e.g. low risk vs high risk studies, random effect model vs fixed effects model). In contrast, subgroup analyses involve comparisons across the different characteristics of the PICO of the primary studies (e.g. grouping different caffeinated drinks together). Sensitivity analysis helps in checking the sensitivity of the overall conclusions to various limitations of the data, assumptions, and approach to analysis. In a subgroup analysis, all participant data included in the meta-analysis is split into subgroups, according to patient characteristics (such as gender) or trial characteristics (such as geographical location), and a meta-analysis is then performed on one or more of these subsets. Such analyses can be used to investigate sources of heterogeneity (differences between treatment effects from individual trials in the meta-analysis), or to provide estimates of treatment effect for clinically relevant subgroups of patients, i.e. the review authors have reason to believe that treatment effect may vary among different subgroups of patients, perhaps due to results from previously conducted studies. |
| 4.6 Were biases in primary studies minimal or addressed in the synthesis? | 12. If meta-analysis was performed, did the review authors assess the potential impact of RoB in individual studies on the results of the meta-analysis? | Respond "Yes/Probably Yes" if: • All studies having received a “low risk of bias” rating  • When studies were judged at high risk of bias, AND sensitivity or subgroup analyses/ adjustment approaches were used (e.g. high risk and low risk studies are grouped in a separate meta-analysis) • When a GRADE approach was used, and discussed in the results or discussion section  • When subgroup analysis not possible (e.g. too few studies) but they discussed the bias of the studies in the discussion/limitations section  Answer "No/Probably No" if: • You suspect there are important bias in included studies that has been ignored by the review authors, or there is "No information".  • If risk of bias was not assessed AT ALL in the included studies  • If bias was assessed but authors did not incorporated it into discussion/ limitations sections  4.6 is "NO" if there is no information provided as the authors should have done this. |
|  | 10. Did the review authors report on the sources of funding for the studies included in the review? | This is about whether the authors looked at funding sources for the included primary studies (Not the review itself). |
|  | 16. Did the review authors report any potential sources of conflict of interest, including any funding they received for conducting the review? | May be found at the top of the manuscript in the sidelines, or bottom of the manuscript, perhaps under acknowledgements |
| ROBIS Concerns regarding regarding the synthesis and findings (low, high, unclear) |  | Automated so do not change AUTOMATED DO NOT TOUCH – this is an excel macro which is automated  ROBIS:  Low risk: If all 3 items 4.3, 4.4 and 4.6 are Yes/Probably Yes, AND • Item 4.4 the study has not conducted a meta-analysis (response option "No meta-analysis"), then this item is ignored here • Item 4.5 has 9 studies or less and is Not Applicable; or response option "No meta-analysis" it is ignored here • Items 4.1 is No Information or Yes/Probably Yes  • Items 4.2 is No Information or Yes/Probably Yes High risk: If any of the items 4.1,4.1,.2, 4.3, 4.4 and 4.6 are "NO/Probably NO" |
| A. Did the interpretation of findings address all of the concerns identified in Domains 1 to 4? | AMSTAR 13 (one component of the ROBIS item).Did the review authors account for RoB in individual studies when interpreting/ discussing the results of the review? | ROBIS = Did the concerns you identified in each domain (specifically the “No” and “Probably No” judgements) get addressed in the limitations section, or did it get discussed in discussion section? • The domain level text needs to be cross-checked against the limitation section of the review AMSTAR 2 item 13= In the discussion or conclusions, did the authors qualify the findings by discussing risk of bias in the included primary studies? |
| B. Was the relevance of identified studies to the review's research question appropriately considered? |  | Did the PICO of the included primary studies match the PICO of the review? (same response as 1.2)  • A. Was the PICO (specifically population or intervention) of the primary studies listed in the review? If yes, then did they closely match the review PICO?  • If no population or intervention of the primary studies (or the review) was listed then put "No" |
| C. Did the reviewers avoid emphasizing results on the basis of their statistical significance? |  | Here we are concerned there is evidence of "spin". We want there to be a balanced analysis based on all analyses, not just the statistically significant ones. So the results of all outcomes should be discussed.  • Review authors should not describe results as ‘statistically significant’, ‘not statistically significant’ or ‘non-significant’ or unduly rely on thresholds for P values, but report the confidence interval together with the exact P value • Confidence intervals should be reported: A 95% confidence interval is often interpreted as indicating a range within which we can be 95% certain that the true effect lies. The width of the confidence interval for an individual study depends to a large extent on the sample size, and the number of events/range of the means.  https://training.cochrane.org/handbook/current/chapter-15#_Ref524097083 |
| ROBIS Overall Judgment: (low, high, unclear) |  | Automated so do not change - this is an excel macro which is automated  If any of the domains is High risk, the overall judgment is High risk If all domains are low risk then the overall judgment is Low risk |
| AMSTAR 2 overall judgments |  | Automated so do not change - this is an excel macro which is automated |

**Appendix D: Data elements extracted**

**Characteristics**

ICD-10 medical classification, Number of authors per SR, Number of authors per SR, median (IQR), Country of first authors, Continent of first authors, Year of publication, Type of SR synthesis, Type of SR, Number of included primary studies, Number of included primary studies, median (IQR), certainty of evidence approach,

Certainty of evidence ratings, Funding, Conflict of interests declared, Protocol, Language, Equity focused, Update of the SR, Imprecision, Number of databases searched.

**Assessment**

Assessment items from AMSTAR-2 and ROBIS by item and overall rating.

**Appendix E: ROBIS and AMSTAR-2 overall judgments**

**Table E.1: ROBIS and AMSTAR-2 overall judgments, and ROBIS domain-level judgments (n = 200)**

| **Systematic review (SR)** | **ROBIS domains** | | | | **Overall response** | |
| --- | --- | --- | --- | --- | --- | --- |
|  | **1. Study eligibility criteria** | **2. ID and selection of studies** | **3. Data collection and study appraisal** | **4. Synthesis and findings** | **ROBIS** | **AMSTAR-2** |
| **Cochrane SR with meta-analysis (n = 47)** | | | | | | |
| Dalal et al, 2017 | Low risk | Low risk | Low risk | High risk | High risk | Low quality |
| Norman et al, 2017 | Low risk | Low risk | Low risk | Low risk | Low risk | High quality |
| Barbaric et al, 2108 | Low risk | Low risk | Low risk | Low risk | Low risk | High quality |
| Heras-Mosteiro et al, 2017 | Low risk | Low risk | Low risk | Low risk | Low risk | High quality |
| Akl et al, 2014 | Low risk | Low risk | Low risk | Low risk | Low risk | High quality |
| Alikhan et al, 2014 | Low risk | High risk | Low risk | High risk | High risk | Moderate quality |
| Akl et al, 2017 | Low risk | Low risk | Low risk | Low risk | Low risk | High quality |
| Dong et al, 2016 | High risk | Low risk | Low risk | Low risk | High risk | Moderate quality |
| Di Nisio et al, 2016 | High risk | Low risk | High risk | Low risk | High risk | Low quality |
| Hansen et al, 2019 | Low risk | Low risk | Low risk | High risk | High risk | Low quality |
| Stern et al, 2017 | Low risk | Low risk | Low risk | Low risk | Low risk | High quality |
| Karsch-Völk et al, 2014 | High risk | High risk | Low risk | High risk | High risk | Critically low quality |
| Bar-On et al, 2012 | Low risk | Low risk | Low risk | Low risk | Low risk | High quality |
| Jat et al, 2022 | Low risk | Low risk | Low risk | Low risk | Low risk | Low quality |
| Altmann et al, 2019 | High risk | High risk | Low risk | Low risk | High risk | Moderate quality |
| Crossingham et al, 2017 | Low risk | Low risk | Low risk | Low risk | Low risk | High quality |
| Welsh et al, 2010 | High risk | Low risk | Low risk | Low risk | High risk | Moderate quality |
| Cates et al, 2012 | Low risk | Low risk | High risk | Low risk | High risk | High quality |
| Santino et al, 2020 | Low risk | Low risk | Low risk | Low risk | Low risk | High quality |
| Anderson-James et al, 2013 | Low risk | Low risk | Low risk | Low risk | Low risk | High quality |
| Ni et al, 2014 | Low risk | Low risk | Low risk | Low risk | Low risk | High quality |
| Lewis et al, 2018 | Low risk | Low risk | Low risk | Low risk | Low risk | High quality |
| Cakmakkaya et al, 2014 | High risk | High risk | Low risk | Low risk | High risk | Low quality |
| Blessberger et al, 2019 | High risk | Low risk | Low risk | Low risk | High risk | High quality |
| Guay et al, 2015 | High risk | High risk | High risk | High risk | High risk | Moderate quality |
| Bizzarro et al, 2014 | Low risk | Low risk | Low risk | Low risk | Low risk | Moderate quality |
| Wetterslev et al, 2015 | Low risk | High risk | High risk | Low risk | High risk | High quality |
| Madrid et al, 2016 | Low risk | Low risk | Low risk | Low risk | Low risk | High quality |
| Galvin et al, 2015 | High risk | Low risk | Low risk | Low risk | High risk | Moderate quality |
| Chou et al, 2022 | Low risk | Low risk | Low risk | Low risk | Low risk | High quality |
| Furlan et al, 2015 | Low risk | Low risk | Low risk | High risk | High risk | High quality |
| Traeger et al, 2023 | Low risk | Low risk | Low risk | Low risk | Low risk | High quality |
| Mu et al, 2020 | Low risk | Low risk | Low risk | Low risk | Low risk | High quality |
| Oliveira et al, 2020 | Low risk | Low risk | Low risk | Low risk | Low risk | High quality |
| Wieland et al, 2022 | Low risk | Low risk | Low risk | High risk | High risk | High quality |
| Brunskill et al, 2015 | Low risk | Low risk | Low risk | Low risk | Low risk | Low quality |
| Herbert et al, 2011 | Low risk | Low risk | Low risk | Low risk | Low risk | Moderate quality |
| Khan et al, 2010 | Low risk | Low risk | Low risk | High risk | High risk | Moderate quality |
| Goldman et al, 2010 | High risk | Low risk | Low risk | Low risk | High risk | Moderate quality |
| Cameron et al, 2018 | High risk | High risk | Low risk | Low risk | High risk | Moderate quality |
| Lahart et al, 2018 | Low risk | Low risk | Low risk | Low risk | Low risk | High quality |
| Guaiana et al, 2010 | High risk | High risk | Low risk | High risk | High risk | High quality |
| Kisely et al, 2017 | High risk | Low risk | Low risk | Low risk | High risk | Moderate quality |
| Hróbjartsson et al, 2010 | Low risk | Low risk | Low risk | High risk | High risk | Moderate quality |
| Deshpande et al, 2012 | Low risk | Low risk | Low risk | Low risk | Low risk | High quality |
| Greenhalgh et al, 2021 | High risk | Low risk | Low risk | Low risk | High risk | High quality |
| Cameron et al, 2022 | Low risk | Low risk | Low risk | Low risk | Low risk | Low quality |
| **Cochrane SR without meta-analysis (n = 21)** | | | | | | |
| Gopalakrishnan et al, 2017 | Low risk | Low risk | High risk | High risk | High risk | Critically low quality |
| Ramasubbu et al, 2017 | Low risk | High risk | Low risk | Low risk | High risk | Low quality |
| Pan et al, 2017 | Low risk | Low risk | Low risk | Low risk | Low risk | High quality |
| Chandelia et al, 2020 | Low risk | Low risk | Low risk | Low risk | Low risk | High quality |
| Galani et al, 2014 | Low risk | Low risk | Low risk | Low risk | Low risk | High quality |
| Hayward et al, 2015 | Low risk | Low risk | Low risk | Low risk | Low risk | High quality |
| Gardiner et al, 2015 | Low risk | Low risk | Low risk | Low risk | Low risk | High quality |
| Lee et al, 2015 | Low risk | Low risk | Low risk | Low risk | Low risk | High quality |
| Korang et al, 2016 | Low risk | Low risk | Low risk | Low risk | Low risk | High quality |
| Hounsome et al, 2016 | Low risk | Low risk | Low risk | Low risk | Low risk | Low quality |
| Murphy et al, 2014 | Low risk | High risk | Low risk | Low risk | High risk | High quality |
| Oltean et al, 2014 | High risk | Low risk | Low risk | Low risk | High risk | High quality |
| Romano et al, 2012 | Low risk | Low risk | Low risk | Low risk | Low risk | Low quality |
| Trinh et al, 2010 | Low risk | Low risk | Low risk | Low risk | Low risk | Low quality |
| Waseem et al, 2011 | Low risk | Low risk | Low risk | Low risk | Low risk | Moderate quality |
| Monk et al, 2016 | High risk | High risk | Low risk | Low risk | High risk | High quality |
| Zhao et al, 2017 | Low risk | Low risk | Low risk | Low risk | Low risk | High quality |
| Goossen et al, 2013 | Low risk | Low risk | Low risk | High risk | High risk | Low quality |
| Broderick et al, 2019 | High risk | High risk | High risk | High risk | High risk | Critically low quality |
| Barnes et al, 2017 | High risk | Low risk | Low risk | Low risk | High risk | Low quality |
| Karkou et al, 2023 | Low risk | Low risk | Low risk | Low risk | Low risk | High quality |
| **Non-Cochrane SR with MA (N= 85)** | | | | | | |
| Jin et al, 2017 | Low risk | High risk | High risk | High risk | High risk | Critically low quality |
| Shi et al, 2017 | High risk | High risk | Low risk | High risk | High risk | Critically low quality |
| Naik et al, 2017 | Low risk | Low risk | Low risk | Low risk | Low risk | Low quality |
| Christensen et al, 2018 | High risk | High risk | High risk | High risk | High risk | Critically low quality |
| Puig et al, 2017 | High risk | High risk | High risk | High risk | High risk | Critically low quality |
| Parker et al, 2017 | High risk | High risk | Low risk | Low risk | High risk | Critically low quality |
| El Hajj et al, 2017 | High risk | High risk | Low risk | Low risk | High risk | Critically low quality |
| Girish et al, 2017 | High risk | High risk | Low risk | High risk | High risk | Critically low quality |
| Zhou et al, 2016 | High risk | High risk | High risk | High risk | High risk | Critically low quality |
| Ungprasert et al, 2016 | High risk | High risk | Low risk | High risk | High risk | Critically low quality |
| Guo et al, 2017 | High risk | High risk | Low risk | Low risk | High risk | Critically low quality |
| Chen et al, 2017 | High risk | High risk | Low risk | High risk | High risk | Critically low quality |
| Aghbari et al, 2017 | High risk | High risk | Low risk | High risk | High risk | Critically low quality |
| Wu et al, 2017 | High risk | High risk | High risk | Low risk | High risk | Critically low quality |
| Singh et al, 2017 | High risk | High risk | High risk | High risk | High risk | Critically low quality |
| Zhang et al, 2017 | Low risk | High risk | High risk | Low risk | High risk | Critically low quality |
| Ruiter et al, 2017 | High risk | High risk | High risk | High risk | High risk | Critically low quality |
| Han et al, 2017 | High risk | High risk | High risk | Low risk | High risk | Critically low quality |
| Chen et al, 2017 | High risk | High risk | High risk | High risk | High risk | Critically low quality |
| Coyle et al, 2017 | Low risk | High risk | High risk | High risk | High risk | Critically low quality |
| Chasset et al, 2017 | High risk | High risk | High risk | High risk | High risk | Critically low quality |
| Zani et al, 2017 | High risk | High risk | High risk | High risk | High risk | Critically low quality |
| Gonzalez-Lopez et al, 2017 | Low risk | Low risk | Low risk | High risk | High risk | Critically low quality |
| Sacco et al, 2017 | High risk | High risk | High risk | High risk | High risk | Critically low quality |
| Shamliyan et al, 2017 | High risk | High risk | High risk | Low risk | High risk | Critically low quality |
| Aoki et al, 2017 | High risk | High risk | High risk | High risk | High risk | Critically low quality |
| Shahwan et al, 2017 | High risk | High risk | High risk | High risk | High risk | Critically low quality |
| Simonsen et al, 2017 | High risk | High risk | High risk | High risk | High risk | Critically low quality |
| Yang et al, 2017 | High risk | High risk | High risk | High risk | High risk | Critically low quality |
| Bae et al, 2017 | High risk | High risk | Low risk | Low risk | High risk | Critically low quality |
| Peng et al, 2017 | Low risk | Low risk | High risk | High risk | High risk | Critically low quality |
| Karia et al, 2017 | High risk | High risk | High risk | High risk | High risk | Critically low quality |
| Upala et al, 2016 | High risk | High risk | High risk | High risk | High risk | Critically low quality |
| Ng et al, 2017 | High risk | High risk | Low risk | Low risk | High risk | Critically low quality |
| Guo et al, 2017 | High risk | High risk | Low risk | Low risk | High risk | Critically low quality |
| Singh et al, 2017 | High risk | Low risk | Low risk | High risk | High risk | Critically low quality |
| Ungprasert et al, 2017 | High risk | High risk | High risk | High risk | High risk | Critically low quality |
| Barbary et al, 2018 | Low risk | High risk | High risk | Low risk | High risk | Critically low quality |
| Ou et al, 2018 | High risk | High risk | High risk | High risk | High risk | Critically low quality |
| Gao et al, 2017 | High risk | High risk | Low risk | High risk | High risk | Critically low quality |
| Jin et al, 2018 | Low risk | High risk | Low risk | High risk | High risk | Critically low quality |
| Pampena et al, 2017 | High risk | High risk | High risk | High risk | High risk | Critically low quality |
| Bui et al, 2018 | High risk | High risk | High risk | High risk | High risk | Critically low quality |
| Rugo et al, 2017 | Low risk | High risk | Low risk | High risk | High risk | Low quality |
| Kuo et al, 2017 | High risk | High risk | Low risk | Low risk | High risk | Critically low quality |
| Wang et al, 2017 | High risk | High risk | Low risk | High risk | High risk | Critically low quality |
| Chen et al, 2017 | Low risk | High risk | High risk | Low risk | High risk | Critically low quality |
| Jørgensen et al, 2017 | Low risk | Low risk | High risk | High risk | High risk | Critically low quality |
| Student et al, 2017 | High risk | High risk | Low risk | High risk | High risk | Critically low quality |
| Checchio et al, 2017 | High risk | High risk | High risk | High risk | High risk | Critically low quality |
| Groen et al, 2017 | Low risk | High risk | High risk | High risk | High risk | Critically low quality |
| Wei et al, 2018 | High risk | Low risk | High risk | High risk | High risk | Critically low quality |
| Gatmaitan-Dumlao et al, 2017 | High risk | High risk | High risk | High risk | High risk | Critically low quality |
| Espinoza et al, 2022 | Low risk | High risk | Low risk | High risk | High risk | Critically low quality |
| Crowley et al, 2017 | Low risk | High risk | Low risk | High risk | High risk | Low quality |
| Hansen et al, 2017 | High risk | High risk | High risk | Low risk | High risk | Low quality |
| Shi et al, 2018 | Low risk | High risk | High risk | Low risk | High risk | Low quality |
| Storgaard et al, 2016 | Low risk | Low risk | High risk | Low risk | High risk | Critically low quality |
| Corcoran et al, 2008 | High risk | High risk | High risk | High risk | High risk | Critically low quality |
| Yu et al, 2016 | High risk | High risk | High risk | High risk | High risk | Critically low quality |
| Lloyd et al, 2008 | High risk | High risk | High risk | High risk | High risk | Critically low quality |
| Tun et al, 2016 | High risk | High risk | High risk | High risk | High risk | Critically low quality |
| Randolph et al, 1998 | Low risk | High risk | Low risk | High risk | High risk | Critically low quality |
| Sanford et al, 2014 | Low risk | High risk | High risk | High risk | High risk | Critically low quality |
| Zhang et al, 2013 | Low risk | High risk | High risk | High risk | High risk | Critically low quality |
| Själander et al, 2007 | High risk | Low risk | High risk | High risk | High risk | Critically low quality |
| Che et al, 2013 | Low risk | Low risk | High risk | High risk | High risk | Critically low quality |
| Yalamanchili et al, 2005 | High risk | High risk | High risk | High risk | High risk | Critically low quality |
| Bump et al, 2009 | High risk | High risk | High risk | High risk | High risk | Critically low quality |
| Ben-Aharon et al, 2014 | Low risk | High risk | High risk | Low risk | High risk | Critically low quality |
| Bath et al, 2000 | Low risk | Low risk | Low risk | High risk | High risk | Critically low quality |
| Kanaan et al, 2007 | Low risk | High risk | High risk | High risk | High risk | Critically low quality |
| Dentali et al, 2007 | High risk | High risk | Low risk | Low risk | High risk | Low quality |
| Ageno et al, 2015 | High risk | High risk | Low risk | Low risk | High risk | Critically low quality |
| Wein et al, 2007 | Low risk | Low risk | High risk | High risk | High risk | Critically low quality |
| Kirkpatrick et al, 2007 | High risk | High risk | High risk | High risk | High risk | Critically low quality |
| Andre et al, 2007 | High risk | High risk | High risk | High risk | High risk | Critically low quality |
| Mismetti et al, 2000 | Low risk | Low risk | High risk | High risk | High risk | Critically low quality |
| Phan et al, 2014 | High risk | High risk | High risk | Low risk | High risk | Critically low quality |
| Carrier et al, 2007 | High risk | High risk | High risk | High risk | High risk | Critically low quality |
| Chaukiyal et al, 2008 | Low risk | High risk | High risk | High risk | High risk | Critically low quality |
| Lederle et al, 2011 | High risk | High risk | Low risk | High risk | High risk | Critically low quality |
| Kamphuisen et al, 2007 | High risk | High risk | High risk | Low risk | High risk | Critically low quality |
| Gutiérrez Espinoza et al, 2021 | High risk | High risk | High risk | High risk | High risk | Critically low quality |
| **Non-Cochrane SR without meta-analysis (n = 47)** | | | | | | |
| Holm et al, 2017 | High risk | High risk | High risk | High risk | High risk | Critically low quality |
| Berntsen et al, 2017 | Low risk | High risk | High risk | High risk | High risk | Critically low quality |
| Bustan et al, 2017 | High risk | High risk | High risk | High risk | High risk | Critically low quality |
| Bijlard et al, 2017 | High risk | High risk | High risk | High risk | High risk | Critically low quality |
| Dunne et al, 2017 | High risk | High risk | High risk | High risk | High risk | Critically low quality |
| Miguel et al, 2016 | High risk | High risk | High risk | High risk | High risk | Critically low quality |
| Svendsen et al, 2017 | High risk | High risk | High risk | Low risk | High risk | Critically low quality |
| Nasr et al, 2017 | High risk | High risk | High risk | High risk | High risk | Critically low quality |
| Gupta et al, 2017 | Low risk | Low risk | High risk | High risk | High risk | Critically low quality |
| Brown et al, 2017 | High risk | High risk | High risk | High risk | High risk | Critically low quality |
| Xue et al, 2016 | High risk | High risk | High risk | High risk | High risk | Critically low quality |
| Welsh et al, 2017 | High risk | High risk | High risk | High risk | High risk | Critically low quality |
| Borab et al, 2017 | High risk | Low risk | Low risk | High risk | High risk | Critically low quality |
| Barton et al, 2017 | High risk | High risk | High risk | High risk | High risk | Critically low quality |
| Pather et al, 2017 | High risk | High risk | Low risk | High risk | High risk | Moderate quality |
| Alderden et al, 2017 | High risk | High risk | High risk | Low risk | High risk | Critically low quality |
| Nguyen et al, 2017 | High risk | High risk | High risk | High risk | High risk | Critically low quality |
| Nghiem et al, 2017 | High risk | High risk | High risk | Low risk | High risk | Critically low quality |
| Chen et al, 2017 | High risk | High risk | High risk | High risk | High risk | Critically low quality |
| Robert et al, 2017 | Low risk | High risk | High risk | High risk | High risk | Critically low quality |
| Hsu et al, 2017 | High risk | High risk | High risk | High risk | High risk | Critically low quality |
| Kolkhir et al, 2017 | High risk | High risk | High risk | High risk | High risk | Critically low quality |
| Andrades et al, 2017 | Low risk | High risk | High risk | High risk | High risk | Critically low quality |
| Shih et al, 2017 | High risk | High risk | High risk | High risk | High risk | Critically low quality |
| Pirone et al, 2017 | Low risk | High risk | Low risk | Low risk | High risk | Critically low quality |
| Snast et al, 2017 | High risk | High risk | High risk | High risk | High risk | Critically low quality |
| Jabbour et al, 2017 | High risk | High risk | High risk | High risk | High risk | Critically low quality |
| Mendizábal et al, 2017 | High risk | High risk | High risk | High risk | High risk | Critically low quality |
| DeJong et al, 2017 | High risk | High risk | High risk | Low risk | High risk | Critically low quality |
| Liu et al, 2017 | High risk | High risk | High risk | Low risk | High risk | Critically low quality |
| Bitan et al, 2017 | High risk | High risk | High risk | High risk | High risk | Critically low quality |
| Buggy et al, 2017 | High risk | High risk | High risk | High risk | High risk | Critically low quality |
| Eskeland et al, 2017 | High risk | High risk | High risk | High risk | High risk | Critically low quality |
| Spring et al, 2017 | Low risk | High risk | Low risk | Low risk | High risk | Critically low quality |
| Mahmoud, 2017 | High risk | High risk | High risk | Low risk | High risk | Critically low quality |
| Blaizot et al, 2017 | High risk | High risk | High risk | High risk | High risk | Critically low quality |
| Picard et al, 2017 | High risk | High risk | High risk | Low risk | High risk | Critically low quality |
| Weidner et al, 2017 | Low risk | Low risk | Low risk | High risk | High risk | Critically low quality |
| Vallerand et al, 2017 | High risk | Low risk | High risk | High risk | High risk | Critically low quality |
| Peleva et al, 2017 | High risk | High risk | High risk | High risk | High risk | Low quality |
| Nguyen et al, 2017 | High risk | High risk | High risk | High risk | High risk | Critically low quality |
| Wong et al, 2018 | High risk | High risk | High risk | High risk | High risk | Critically low quality |
| Hald et al, 2017 | High risk | High risk | High risk | High risk | High risk | Critically low quality |
| Co et al, 2017 | High risk | High risk | High risk | High risk | High risk | Critically low quality |
| Guarana et al, 2018 | High risk | High risk | High risk | High risk | High risk | Critically low quality |
| Fortuna et al, 2017 | Low risk | Low risk | Low risk | High risk | High risk | Critically low quality |
| Black et al, 2017 | High risk | High risk | Low risk | Low risk | High risk | Low quality |
| Price et al, 2015 | High risk | High risk | High risk | High risk | High risk | High quality |

***Note:*** *Salmon highlighting indicates disagreement in direction of rating between ROBIS and AMSTAR-2 overall response judgments****.***

**Appendix F: Comparison of ROBIS and AMSTAR-2 item ratings**

**Table F.1: ROBIS items assessed by Cochrane and non-Cochrane systematic SRs (n=200)**

| **Items** | **Total SRs**  **n (%)** | **Cochrane SRs**  **(n = 68)** | | **Non-Cochrane SRs**  **(n = 132)** | |
| --- | --- | --- | --- | --- | --- |
|  |  | **Pairwise meta-analysis**  **(n = 47)** | **No meta-analysis**  **(n = 21)** | **Pairwise meta-analysis**  **(n = 85)** | **No meta-analysis**  **(n = 47)** |
| 1.1 Did the SR adhere to pre-defined objectives and eligibility criteria? | 89 (44.5%) | 47 (100.0%) | 21 (100.0%) | 12 (14.3%) | 9 (18.8%) |
| 1.2 Were the eligibility criteria appropriate for the SR question? | 192 (96.0%) | 46 (97.9%) | 21 (100.0%) | 81 (95.3%) | 44 (93.6%) |
| 1.3 Were eligibility criteria unambiguous? | 171 (85.5%) | 47 (100.0%) | 21 (100.0%) | 75 (88.2%) | 29 (61.7%) |
| 1.4 Were all restrictions in eligibility criteria based on date, sample size, study quality, outcomes measured appropriate? | 121 (60.5%) | 37 (78.7%) | 19 (90.5%) | 44 (51.8%) | 24 (51.1%) |
| 1.5 Were any restrictions in eligibility criteria based publication status or format, language, availability of data appropriate? | 91 (45.5%) | 35 (74.5%) | 15 (72.4%) | 30 (35.3%) | 11 (23.4%) |
| 2.1 Did the search include an appropriate range of databases/ electronic sources for published and unpublished reports? | 156 (78%) | 47 (100.0%) | 21 (100.0%) | 64 (76.2%) | 24 (51.1%) |
| 2.2 Were methods additional to database searching used to identify relevant reports? | 162 (81.0%) | 46 (97.9%) | 20 (95.2%) | 66 (77.6%) | 30 (64.6%) |
| 2.3 Were the terms and structure of the search strategy likely to retrieve as many eligible studies as possible? | 115 (57.5%) | 46 (97.9%) | 20 (95.2%) | 31 (36.9%) | 18 (37.5%) |
| 2.4 Were search strategy restrictions based on date, publication format, or language appropriate? | 111 (55.5%) | 38 (80.91700%) | 17 (81.0%) | 41 (48.2%) | 15 (31.9%) |
| 2.5 Were efforts made to minimise error in selection of studies? | 148 (74%) | 43 (91.5%) | 21 (100.0%) | 59 (69.4%) | 25 (53.2%) |
| 3.1 Were efforts made to minimise error in data collection? | 149 (74.5%) | 45 (95.7%) | 21 (100.0%) | 64 (75.3%) | 19 (40.4%) |
| 3.2 Were sufficient study characteristics considered for both SR authors and readers to be able to interpret the results? | 172 (86.0%) | 47 (100.0%) | 21 (100.0%) | 70 (82.4%) | 34 (72.3%) |
| 3.3 Were all relevant study results collected for use in the synthesis? | 166 (83%) | 46 (97.9%) | 19 (90.5%) | 66 (77.4%) | 35 (74.5%) |
| 3.4 Was risk of bias (or methodological quality) formally assessed using appropriate criteria? | 127 (63.5%) | 46 (97.9%) | 19 (90.5%) | 50 (59.5%) | 12 (25.5%) |
| 3.5 Were efforts made to minimise error in risk of bias assessment? | 116 (57.5%) | 44 (93.6%) | 21 (10041%) | 41 (48.2%) | 10 (21.3%) |
| 4.1 Did the synthesis include all studies that it should? | 180 (90.0%) | 45 (95.7%) | 19 (90.5%) | 74 (87.1%) | 42 (89.4%) |
| 4.2 Were all pre-defined analyses reported or departures explained? | 81 (40.5%) | 44 (93.6%) | 19 (90.5%) | 11 (13.1%) | 7 (14.6%) |
| 4.3 Was the synthesis appropriate given the nature and similarity in the research questions, study designs and outcomes across included studies? | 175 (87.5%) | 46 (97.9%) | 20 (95.2%) | 73 (85.9%) | 36 (76.6%) |
| 4.4 Was between-study variation (heterogeneity) minimal or addressed in the synthesis? | 163 (81.5%) | 44 (93.6%) | 20 (95.2%) | 62 (72.9%) | 37 (78.7%) |
| 4.5 Were the findings robust, e.g., as demonstrated through funnel plot or sensitivity analyses? | 51^a^ (68.9%) | 21^b^ (70.0%) | N/A | 30^c^ (68.2%) | N/A |
| 4.6 Were biases in primary studies minimal or addressed in the synthesis? | 108 (54.0%) | 44 (93.6%) | 18 (85.7%) | 38 (45.2%) | 8 (17.0%) |
| A. Did the interpretation of findings address all of the concerns identified in Domains 1 to 4? | 86 (43.0%) | 44 (93.6%) | 16 (76.2%) | 21 (25%) | 5 (10.6%) |
| B. Was the relevance of identified studies to the SR's research question appropriately considered? | 179 (89.5%) | 46 (97.9%) | 20 (95.2%) | 71 (83.3%) | 44 (89.4%) |
| C. Did the reviewers avoid emphasising results on the basis of their statistical significance? | 154 (77.0%) | 41 (87.2%) | 17 (81.0%) | 63 (75%) | 33 (72.2%) |

***Note:*** *Grey highlighting indicates an item matched to an AMSTAR-2 item.* ***a)*** *The percentage is calculated from 74 SRs which reported meta-analysis with 10 or more included primary studies.* ***b)*** *The percentage is calculated from 30 SRs which included 10 or more primary studies.* ***c)*** *The percentage is calculated from 44 SRs which included 10 or more primary studies.* ***Abbreviations:*** *N/A, not applicable; SR, systematic review.*

**Table F.2: AMSTAR-2 items assessed by Cochrane and non-Cochrane systematic SRs (n=200)**

| **Items** | **Total SRs (n = 200)**  **n (%)** | **Cochrane SRs**  **(n = 68)** | | **Non-Cochrane SRs**  **(n = 132)** | |
| --- | --- | --- | --- | --- | --- |
|  |  | **Pairwise meta-analysis**  **(n = 47)** | **No meta-analysis**  **(n = 21)** | **Pairwise meta-analysis**  **(n = 85)** | **No meta-analysis**  **(n = 47)** |
| 1. Did the research questions and inclusion criteria include the components of PICO? | 170 (84%) | 47 (100.0%) | 21 (100.0%) | 74 (87.1%) | 28 (59.6%) |
| 2. Were methods established prior and did the report justify any significant deviations from the protocol? | 88 (44.0%) | 47 (100.0%) | 20 (95.2%) | 12 (14.1%) | 9 (19.1%) |
| 3. Did the authors explain their selection of the study designs for inclusion in the SR? | 35 (17.5%) | 14 (29.8%) | 5 (28.6%) | 9 (10.6%) | 7 (14.9%) |
| 4. Did the authors use a comprehensive literature search strategy? | 154 (77%) | 47 (100.0%) | 21 (100.0%) | 62 (72.9%) | 24 (51.1%) |
| 5. Did the authors perform study selection in duplicate? | 149 (74.5%) | 44 (93.6%) | 21 (100.0%) | 59 (69.4%) | 25 (53.2%) |
| 6. Did the authors perform data extraction in duplicate? | 149 (74.5%) | 44 (93.6%) | 21 (100.0%) | 64 (75.3%) | 20 (42.6%) |
| 7. Did the authors provide a list of excluded studies and justify the exclusions? | 81 (40.5%) | 46 (97.9%) | 20 (95.2%) | 10 (11.8%) | 5 (10.6%) |
| 8. Did the authors describe the included studies in adequate detail? | 171 (85.5%) | 47 (100.0%) | 21 (100.0%) | 69 (81.2%) | 34 (72.3%) |
| 9.Did the authors use a satisfactory technique for assessing the risk of bias (RoB) in individual studies that were included in the SR? | 127 (63.5%) | 46 (97.9%) | 19 (90.5%) | 50 (58.8%) | 12 (25.5%) |
| 10. Did the authors report on the sources of funding for the studies included in the SR? | 66 (33.0%) | 33 (70.2%) | 14 (66.7%) | 13 (15.3%) | 6 (12.8%) |
| 11.If meta-analysis was performed did the authors use appropriate methods for statistical combination of results? | 118^a^ (89.4%) | 46 (97.9%) | N/A | 72 (84.7%) | N/A |
| 12. If meta-analysis was performed, did the authors assess the potential impact of RoB in individual studies on the results of the meta-analysis? | 80^a^ (60.6%) | 44 (93.6%) | N/A | 36 (42.4%) | N/A |
| 13. Did the authors account for RoB in individual studies when interpreting/ discussing the results of the SR? | 95 (47.5%) | 47 (100.0%) | 17 (81.0%) | 25 (29.4%) | 6 (12.8%) |
| 14. Did the authors provide a satisfactory explanation for, and discussion of, any heterogeneity observed in the results of the SR? | 104^a^ (52%) | 45 (95.7%) | N/A | 59 (69.4%) | N/A |
| 15.If they performed quantitative synthesis did the authors carry out an adequate investigation of publication bias (small study bias) and discuss its likely impact on the results of the SR? | 81^a^ (40.5%) | 39 (78.7%) | N/A | 42 (47.1%) | N/A |
| 16. Did the authors report any potential sources of conflict of interest, including any funding? | 182 (91.0%) | 47 (100.0%) | 21 (100.0%) | 72 (84.7%) | 42 (89.4%) |

***Note:*** *Grey highlighting indicates an item matched to an AMSTAR-2 item.* ***a)*** *The percentage is calculated from 132 SRs which reported meta-analysis.* ***Abbreviations:*** *N/A, not applicable.*
